# Revisiting Netrin-1: One Who Guides (Axons)

**DOI:** 10.3389/fncel.2018.00221

**Published:** 2018-07-31

**Authors:** Nicholas P. Boyer, Stephanie L. Gupton

**Affiliations:** ^1^Neurobiology Curriculum, The University of North Carolina at Chapel Hill, Chapel Hill, NC, United States; ^2^Department of Cell Biology and Physiology, The University of North Carolina at Chapel Hill, Chapel Hill, NC, United States; ^3^Neuroscience Center, The University of North Carolina at Chapel Hill, Chapel Hill, NC, United States

**Keywords:** netrin-1, DCC, UNC5, axon guidance, growth cone, chemotaxis, haptotaxis

## Abstract

Proper patterning of the nervous system requires that developing axons find appropriate postsynaptic partners; this entails microns to meters of extension through an extracellular milieu exhibiting a wide range of mechanical and chemical properties. Thus, the elaborate networks of fiber tracts and non-fasciculated axons evident in mature organisms are formed via complex pathfinding. The macroscopic structures of axon projections are highly stereotyped across members of the same species, indicating precise mechanisms guide their formation. The developing axon exhibits directionally biased growth toward or away from external guidance cues. One of the most studied guidance cues is netrin-1, however, its presentation *in vivo* remains debated. Guidance cues can be secreted to form soluble or chemotactic gradients or presented bound to cells or the extracellular matrix to form haptotactic gradients. The growth cone, a highly specialized dynamic structure at the end of the extending axon, detects these guidance cues via transmembrane receptors, such as the netrin-1 receptors deleted in colorectal cancer (DCC) and UNC5. These receptors orchestrate remodeling of the cytoskeleton and cell membrane through both chemical and mechanotransductive pathways, which result in traction forces generated by the cytoskeleton against the extracellular environment and translocation of the growth cone. Through intracellular signaling responses, netrin-1 can trigger either attraction or repulsion of the axon. Here we review the mechanisms by which the classical guidance cue netrin-1 regulates intracellular effectors to respond to the extracellular environment in the context of axon guidance during development of the central nervous system and discuss recent findings that demonstrate the critical importance of mechanical forces in this process.

## Axon Response to the Environment

Development of an animal nervous system, from that of the nematode *Caenorhabditis elegans* to larger mammals such as humans, requires that each neuron connect to proper target cells. This is accomplished by the extension of a specialized projection, the axon, from the neuronal cell body. The growing axon traverses relatively long distances, up to several thousand times the diameter of the cell body, and reaches the correct region to produce the stereotyped circuits found across members of a species. A complex, cytoskeletal rich structure at the end of a developing axon, the growth cone, is responsible for not only extending the axon, but also detecting and responding to the extracellular signals that direct pathfinding. These signals, frequently in the form of glycoproteins secreted into or presented attached to the extracellular matrix, are ligands for receptors on the surface of the growth cone and trigger a variety of intracellular responses, including membrane remodeling through exocytosis and endocytosis, cytoskeletal reorganization, and modification of protein expression and degradation, both locally in the axon and throughout the neuron. For thorough, recent reviews on growth cone regulation, see “Regulation of plasma membrane expansion during axon formation” on membrane remodeling and addition (Quiroga et al., 2017), “Actin based growth cone motility and guidance” on actin responses in the growth cone (Omotade et al., 2017), “Mechanochemical regulation of growth cone motility” on mechanosensation and mechanotransduction by growth cones (Kerstein et al., 2015), and “Axon Guidance Pathways and the Control of Gene Expression” on regulation of gene expression (Russell and Bashaw, 2018). This review will specifically focus on the mechanisms by which the guidance molecule netrin-1 produces axon guidance responses.

## Netrin-1, The Classical Guidance Cue

**FIGURE 1:**
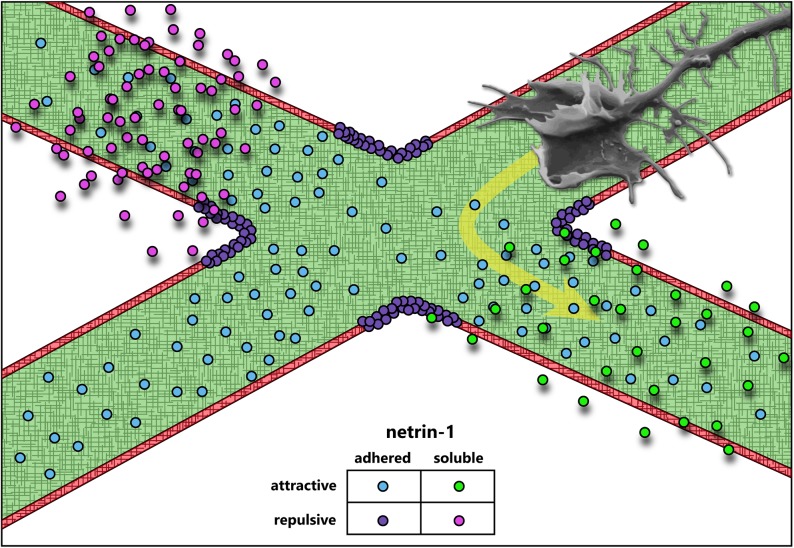
Axon guidance cues attract or repulse growth cones, and can be presented adhered to the extracellular matrix or soluble. The data accumulated over the last 30 years suggest that netrin-1 acts both as an attractive and repulsive cue, and may function both as a soluble, chemotactic cue and as a substrate-adhered, haptotactic cue.

One of the defining discoveries in the field of axon guidance demonstrated that axonal outgrowth promoted by a previously unknown and presumably diffusible extracellular cue, was biased in the direction of the cue source (Serafini et al., 1994). Purification of the major factors from chick brain that promoted axon outgrowth in embryonic rat spinal cord explants yielded two proteins homologous to the *C. elegans unc-6* gene product required for axon guidance (Hedgecock et al., 1990), which were named netrin-1 and netrin-2 after the Sanskrit “netr” meaning “one who guides” (Serafini et al., 1994). Further work would show that these were indeed axonal guidance cues (Kennedy et al., 1994; Deiner et al., 1997; Mehlen and Rama, 2007; Moore et al., 2007; Masuda et al., 2009; Xu et al., 2010; Sun et al., 2011), and netrin-1 has since been one of the most well-studied members of this class of proteins with roles in not only axon guidance, but also axon branching (Dent et al., 2004), synaptogenesis (Flores, 2011), cell migration (Ylivinkka et al., 2016), cell survival (Mehlen and Furne, 2005), and axon regeneration (Dun and Parkinson, 2017). This review, however, focuses on the function of netrin-1 as an axon guidance cue. Axon guidance by netrin-1 has been implicated in multiple developing brain regions and developing neuronal types, making it one of the most characterized, diversely functioning guidance cues. Fascinatingly, whereas many axon guidance cues have been found to act predominantly as either attractive or repulsive, and as either diffusible/chemotactic or adhesive/haptotactic molecules, evidence of the function of netrin-1 has never placed it squarely into one category (**Figure 1**). This diversity in function renders netrin-1 an ideal candidate for studies on mechanotransduction in axon guidance, as recent studies have emphasized the importance of substrate adhesion in netrin-1 function *in vivo* (Dominici et al., 2017; Varadarajan et al., 2017; Yamauchi et al., 2017).

### Attraction and Repulsion

**FIGURE 2:**
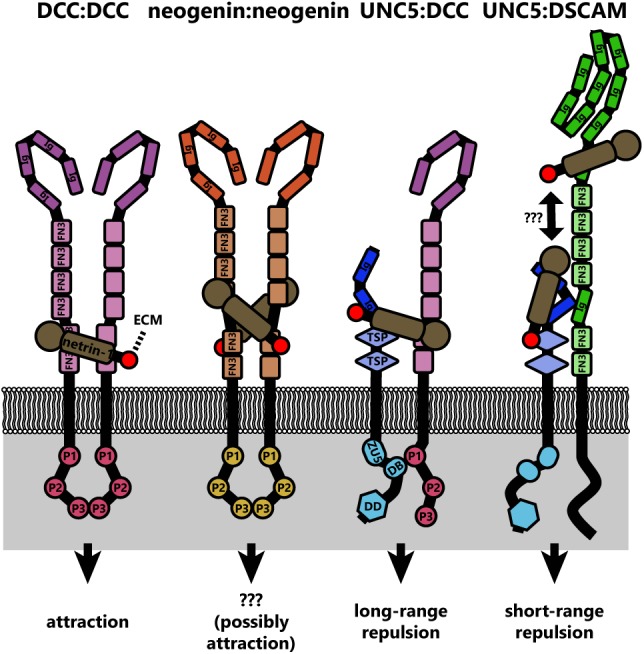
Diagrams of the known netrin-1 receptor dimers and their signaling functions. Netrin-1 binding simultaneously to two DCC molecules induces DCC homodimers, which similarly occurs for neogenin. If netrin is substrate bound, this could crosslink DCC dimers to the extracellular matrix. However, the signaling result of neogenin dimers is not currently known. Whether netrin-1 also links neogenin:neogenin, UNC5:DCC, or UNC5:DSCAM dimers to the extracellular matrix is also unknown, but represents potential mechanotransductive mechanisms in netrin-1 signaling. Structural studies have not been conducted on the UNC5:DSCAM dimer, therefore whether a single netrin-1 molecule binds both receptors is unknown.

Even from the earliest description of the *C. elegans* genes *unc-5* (*UNC5* in mammals), *unc-6* (*NTN1* in mammals), and *unc-40* (*DCC* and *NEO* in mammals, *frazzled* in *Drosophila*), data suggested that a ventral source of *unc-6* both attracts and repulses axons (Hedgecock et al., 1990). Deletion of *unc-6* affects guidance of axons that extend dorsally (repulsion) or ventrally (attraction). Dorsal guidance is specifically impaired by deletion of *unc-5* and ventral guidance is impaired by deletion of *unc-40*, suggesting the different responses are orchestrated by distinct receptors. Later experiments using chick spinal explants describe a bimodal axon outgrowth response to increasing concentrations of netrin-1 (Serafini et al., 1994), with the highest concentrations promoting less robust outgrowth. A concentration-dependent bimodal response is also observed in the turning of embryonic cortical murine axons in a stable gradient of netrin-1 *in vitro* (Taylor et al., 2015). Elegant experiments by a number of labs over the years have established that this bifunctionality of netrin-1 signaling is dependent upon the receptors presented by the axonal growth cone. Netrin binding to the receptor deleted in colorectal cancer (DCC) results in attractive responses, via homodimerization of DCC (covered in detail in later sections) (Chan et al., 1996; Keino-Masu et al., 1996; Kolodziej et al., 1996; Fazeli et al., 1997), whereas heterodimerization between DCC and receptor uncoordinated locomotion 5 (UNC5) converts this attractive response into repulsion (Hamelin et al., 1993; Colavita and Culotti, 1998; Finci et al., 2014). Intriguingly, UNC5 can also mediate shorter-range repulsive responses to netrin-1 in the absence of DCC (Keleman and Dickson, 2001). This repulsive response requires association between UNC5 and the co-receptor, down syndrome cell adhesion molecule (DSCAM) (Purohit et al., 2012). The structures and outcomes of known netrin-1 receptor dimers are summarized in **Figure 2**.

An important feature of many netrin-1 signaling pathways, both attractive and repulsive, appears to be interaction between the cytoplasmic domains of dimerized receptors (**Figure 2**). Netrin-1 induces homodimerization of DCC, bringing their cytoplasmic tails into close proximity (Finci et al., 2014). The close apposition of these domains is thought to create an assembly platform for the association of further signaling effectors. In the case of netrin-induced repulsion, the association of the intracellular P1 domain of DCC and a DCC-binding domain of UNC5 is also required (**Figure 2**) (Hong et al., 1999). Whether netrin-1 repulsion by an UNC5/DSCAM complex analogously involves association between intracellular domains of these two receptors remains to be determined. Though the repertoire of receptors that govern the attractive or repulsive responses to netrin-1 have been identified, the differences in intracellular signaling and mechanotransduction responses between these two modes are comparatively less well understood.

#### Attraction: Repulsion Switching

**FIGURE 3:**
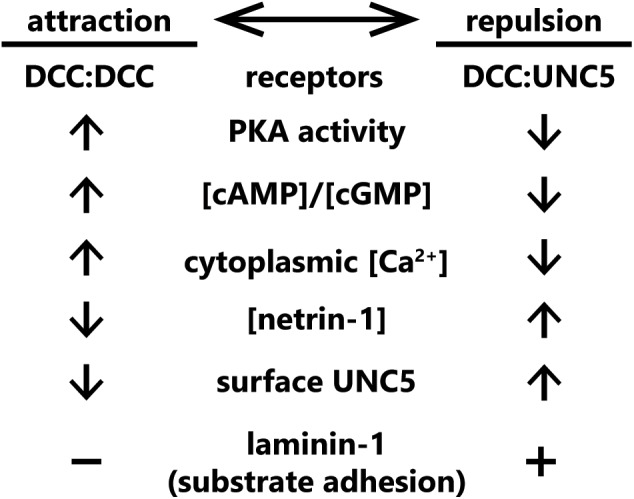
Summary of known “switches” between attractive and repulsive function of netrin-1. Switches span from receptor concentrations at the plasma membrane, cytoplasmic second messenger status, extracellular netrin-1 concentrations, and the presence of other extracellular cues.

These experiments leave us with a glaring question: what determines whether exposure to netrin-1 results in attraction or repulsion of an axon? There are several possibilities for this “switch”; (1) the relative levels of each netrin-1 receptor on the surface of the growth cone; (2) a secondary signal of intracellular status (such as Ca^2+^, cGMP, or cAMP) that may activate or inhibit signaling pathway components, favoring either attraction or repulsion; (3) the relative affinities of individual receptor types for netrin-1, along with the extracellular concentration of netrin-1; or (4) the presence of other molecules in the extracellular environment. Current evidence suggests that all four of these mechanisms are capable of switching netrin-1 responses between attraction and repulsion. Known mechanisms that convert such netrin-1 responses are summarized in **Figure 3**.

#### Switch 1: Membrane Receptor Levels

Modulation of receptor expression levels or presentation on the surface of cells is a common mechanism for tuning responses to extracellular ligands (Groc and Choquet, 2006; Winckler and Yap, 2011; Ceresa, 2012; Hausott and Klimaschewski, 2016; Suh et al., 2018). Altered expression levels and/or surface levels of netrin receptors have also been implicated in modulating response to netrin-1. For example, the DNA repair gene *Rad51* upregulates expression of UNC5B and UNC5C in mouse primary cortical neurons, and negatively regulates netrin-dependent axon branching (Glendining et al., 2017), typically viewed as an attractive response to netrin. Opposing this, endocytic internalization and membrane depletion of UNC5, and not DCC, converts repulsive netrin-1 responses to attraction. This UNC5 internalization is triggered by protein interacting with C-kinase 1 (PICK1)-dependent recruitment of active protein kinase Cα (PKCα) to the plasma membrane, which phosphorylates UNC5 residues S408 and S587 (Williams et al., 2003; Bartoe et al., 2006). Multiple studies suggest that surface levels of DCC are altered by exposure to netrin-1, however, the specific response varies between studies. In embryonic rat spinal commissural and cortical neurons, exposure to netrin-1 increases DCC localization to the plasma membrane (Matsumoto and Nagashima, 2010) potentiated by, but not requiring, protein kinase A (PKA) activation (Bouchard et al., 2004, 2008). A single study in dissociated rat embryonic cortical neurons suggests that DCC is ubiquitinated, internalized, and then degraded after netrin-1 exposure (Kim et al., 2005). However, whether this affects subsequent netrin-1 responses was not shown. Intriguingly KCl-induced depolarization of cortical neurons, but not spinal commissural neurons, leads to increased plasma membrane levels of DCC and increases axon outgrowth in response to netrin-1. These responses require activity of PKA, PKC, and phosphatidylinositol-3-kinases (PI3Ks), as well as exocytosis, but not protein synthesis (Bouchard et al., 2008). Additional studies are required to define how expression levels, surface localization, and protein stability of DCC are modulated by netrin in diverse cell types, and in scenarios in which netrin is perceived by the growth cone as attractive or repulsive.

#### Switch 2: Intracellular Secondary Messengers

Intracellular levels of the secondary messenger, cAMP, which promotes the activity of PKA, may trigger an attractive-repulsive switch in response to netrin. Inhibition of PKA in *Xenopus* spinal neurons with small molecules KT5720 or Rp-cAMPS causes a typically attractive netrin-1 gradient to repulse axons (Ming et al., 1997). The authors conducted a dose-response experiment using Rp-cAMPS, a potent and specific competitive inhibitor of cAMP-dependent activation of PKA, to investigate whether the change from netrin-1 attraction to repulsion was “switch” or a “dial.” This revealed that the turning response transitions abruptly between 1 and 5 μM Rp-cAMPS, rapidly plateauing at higher concentrations: such a sigmoidal response suggests a switch-like mechanism (Ming et al., 1997). Later experiments found that PKA inhibition with a considerably higher dose of KT5720 reduces attractive responses to netrin-1 in rat spinal commissural neurons in a spinal explant, but does not switch the response to repulsion (Moore and Kennedy, 2006). The differences in experimental conditions make interpretation of these data difficult, however, they imply that either higher concentration of PKA inhibitor, some non-neuronal component of the spinal explant, or differences between rat and frog spinal neurons, such as potential differing expression of netrin-1 receptor isoforms, alters the role of PKA in the netrin-1 attraction/repulsion switch.

Netrin-1 response is modulated by other secondary messengers in addition to cAMP/PKA. Axon guidance experiments in isolated *Xenopus* spinal commissural neurons revealed that attractive netrin-1 response relied on both Ca^2+^ release from the endoplasmic reticulum and Ca^2+^ influx through the plasma membrane, and that blockade of Ca^2+^ influx into the cytoplasm converted the attractive response to repulsion (Hong et al., 2000). Ca^2+^-dependent repulsion also requires cGMP. Using cyclic nucleotide analogs, Nishiyama et al. (2003) found that the ratio of [cAMP]/[cGMP] tunes netrin-dependent turning responses; with a high ratio promoting attraction and a low ratio leading to repulsion. Revisiting these classic experiments on netrin-1 attraction and repulsion with new tools to optogenetically manipulate the spatial and temporal distribution of secondary messenger levels is warranted.

#### Switch 3: Netrin-1 Concentration

**FIGURE 4:**
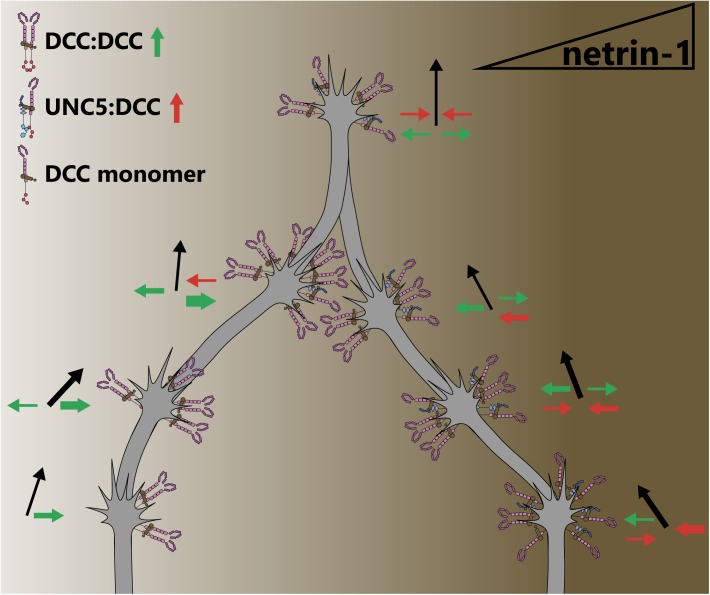
The concentration of netrin-1 may switch attraction to repulsion responses. Attractive and repulsive forces generated by DCC and UNC5 dimers are represented as green and red arrows, respectively, leading to an axon outgrowth vector (black arrows). As the concentration of netrin-1 begins to increase, more DCC homodimers are recruited. At higher netrin-1 concentrations, the promiscuous receptor binding site on netrin begins to recruit UNC5 to dimers, as this binding site has a lower affinity for UNC5 than for DCC. At the highest concentrations of netrin-1, saturation of receptors with ligand may result in receptors being maintained as monomers as opposed to dimerization, preventing downstream signaling that requires a dimer. By virtue of the concentration dependence of attraction and repulsion responses, axons are guided into a concentration in which forces are balanced. However, this may not be a stable position as receptor trafficking, intracellular secondary messengers and extracellular conditions can alter growth cone sensitivity to netrin-1.

Many axon guidance or outgrowth studies reveal bimodal responses of axons occur with increasing netrin concentrations (Serafini et al., 1994; Deiner et al., 1997; De La Torre et al., 1997; Ming et al., 1997; Moore and Kennedy, 2006), suggesting that the concentration of netrin-1 may determine which receptors, and thus intracellular pathways, are recruited. Indeed in a microfluidically isolated gradient of netrin-1, embryonic murine cortical axons closer to the source of netrin-1 (higher concentration) are repelled, whereas those at the lower end of the concentration gradient are attracted (Taylor et al., 2015), supporting the notion of a concentration dependent response. Although further work is needed to establish this mechanism, biophysical experiments have demonstrated that netrin-1 binds with higher affinity to DCC than to UNC5 (Finci et al., 2014). This could lead to increased UNC5/DCC heterodimerization at higher concentrations of netrin-1, inducing repulsive axon guidance responses (Hamelin et al., 1993; Colavita and Culotti, 1998; Finci et al., 2014). The attractive and repulsive forces leading to directional axon outgrowth under this paradigm are summarized in **Figure 4**. Structural studies on the interaction between netrin-1, DCC and UNC5 suggest that although DCC can bind two sites on netrin-1, only one of these can interact with UNC5 (Finci et al., 2014), which would preclude the formation of UNC5 homodimers. Therefore UNC5-dependent, DCC-independent short-range axon repulsion in response to netrin-1 may require dimerization with additional receptors such as DSCAM, discussed later in this review. Alternatively, high concentrations of netrin-1 may saturate DCC, and prevent DCC homodimerization and attractive responsiveness.

#### Switch 4: Extracellular Environment

Surprisingly little is known regarding modification of netrin-1 attraction and repulsion by the composition of the extracellular environment. One other axon guidance molecule, draxin, has been shown to antagonize netrin-1 and is proposed to render netrin-1 a fasciculation cue (Gao et al., 2015; Liu et al., 2018), however, any role in mediating netrin-1 attraction or repulsion is unknown. Additionally, netrin-1 is known to bind to glycosaminoglycans (GAGs), a major component of the extracellular matrix, yet once again the role of these glycoproteins in axon guidance is unknown. Draxin and GAGs will be discussed in more detail later in this review. The integrin ligand laminin-1 is one of the few known extracellular components that switches netrin-1 dependent attraction to repulsion. *Xenopus* retinal growth cones, normally attracted to netrin-1, will be repulsed if laminin-1 is present in the extracellular matrix (ECM) or substrate (Höpker et al., 1999). This response is also likely sensitive to secondary messengers, as in the presence of substrate-adhered laminin-1, blockade of Ca^2+^ release, which is dependent on Ca^2+^-calmodulin-dependent protein kinase II (CaMKII), calcineurin (CaN) and protein phosphatase 1 (PP1), switches a repulsive netrin-1 response to attraction in *Xenopus* spinal neurons (Wen et al., 2004). This suggests an intriguing modulation of internal signaling by extracellular matrix components; indeed in chick ciliary ganglion neurons, treatment with laminin induces an influx of Ca^2+^ (Bixby et al., 1994). Whereas a Ca^2+^ influx blockade can switch netrin-1 attraction to repulsion (Hong et al., 2000), a further increase of Ca^2+^ beyond the attractive netrin-1 response regime may switch the response once more. The integration of signaling events from a complex extracellular environment represents a rich area for future studies into axon guidance. The requirement for substrate adhesion of laminin-1 may also indicate that the netrin-1 attractive-repulsive switch is in part reliant on mechanotransduction pathways, however, this remains to be investigated. This is an especially intriguing area for study of mechanical regulation of axon guidance, as many mechanotransduction proteins are known to be ion channels, which could modify the intracellular environment (Benavides Damm and Egli, 2014; Piperi and Basdra, 2015; Geng et al., 2017).

### Chemotaxis and Haptotaxis?

The dogma of the field has long posited that netrin-1 is a diffusible cue, supported by assays in which axons extend from explants toward netrin-1 secreted by distant patches of cells (Tessier-Lavigne et al., 1988; Kennedy et al., 1994; Serafini et al., 1994; Yamauchi et al., 2017; Xu et al., 2018) or diffusing from enriched agarose blocks (Xu et al., 2018), and by the presence of a gradient of netrin-1 protein in embryonic chick spinal cord (Kennedy et al., 2006). However, a new axis recently emerged for netrin-1 during haptotactic axon guidance. Netrin-1 binds the extracellular matrix (Kennedy et al., 1994) or cell membranes (Kennedy et al., 2006) and guides axons locally (Deiner et al., 1997; Brankatschk and Dickson, 2006), supporting a potential role for netrin as a haptotactic cue that promotes mechanotransduction. When beads covalently linked to netrin-1 are presented to an extending spinal commissural axon *in vitro*, the growth cone exerts force on the bead (Moore et al., 2009). If the bead is immobilized, growth cones reorient toward the bead. Adhesion of netrin-1 to the substrate is suggested to be necessary for attractive axon guidance in spinal commissural neurons, as inhibiting netrin adhesion with heparin blocks this attractive response, and deletion of the highly positively charged C-terminal extracellular matrix-binding C domain of netrin-1 reduces axon outgrowth (Moore et al., 2012). This attraction to adhesive netrin involves non-muscle myosin II (MyoII)-dependent mechanotransduction, as blebbistatin treatment blocked the generation of forces on netrin-1 beads by the growth cone. Indeed, netrin-1 signaling through DCC activates MyoII via indirect activation of myosin light-chain kinase (MLCK) (Murray et al., 2010). MyoII also promotes mechanical activation of focal adhesion kinase (FAK), an important downstream effector of netrin-1 signaling through DCC (Moore et al., 2012) (covered in more detail in a later section). With these several pieces of evidence supporting a haptotactic response to netrin-1, now the relative contributions of haptotactic vs. chemotactic responses to netrin need to be revisited. Fragments of netrin-1 that lack what are considered the major ECM-binding domains are able to produce axon outgrowth responses (Moore et al., 2012) despite reduced interaction with the substrate, suggestive of potential adhesion-independent, chemotactic effects of netrin-1 on the axon. Whether these fragments maintain residual substrate binding that mediates haptotactic axon reorientation remains to be shown.

The original studies on the function of netrin-1 *in vivo* relied on a hypomorphic gene trap allele of *Ntn1* that maintained low levels of netrin-1 protein (Serafini et al., 1996), however, recent development of mice carrying a floxed *Ntn1* allele has allowed for tissue-specific and complete loss of netrin-1 (Bin et al., 2015; Yung et al., 2015). Three recent papers have galvanized the potential role of netrin-1 as a haptotactic cue *in vivo* by selectively deleting netrin-1 from the floor plate and/or ventricular zone of the spine and hindbrain using floxed *Ntn1* alleles, and assessing the midline crossing of commissural axons (Dominici et al., 2017; Varadarajan et al., 2017; Yamauchi et al., 2017) (for an additional mini-review on Dominici et al. and Varadarajan et al., see Morales, 2018). All three groups found that netrin-1 expression in the floor plate, originally thought to be the source of the attractive gradient of netrin-1 responsible for commissural crossing (Maclennan et al., 1997; Kennedy et al., 2006), is not necessary for commissure formation. Rather, netrin-1 secreted by ventricular zone neural progenitors is deposited on the pial surface and forms a path for axons to reach the site of the commissure (Dominici et al., 2017; Varadarajan et al., 2017). Netrin-1 deposition on the extending axons occurs in a DCC-dependent manner (Varadarajan et al., 2017). Presentation of netrin-1 on these axons could be maintained where it is initially bound by DCC, as the growth cone continues to extend beyond these sites, or alternatively could be distributed down the axon by retrograde transport of netrin-1/DCC from the growth cone (Moore et al., 2009). Although deposition of netrin-1 on a surface supports a haptotactic guidance role, it does not invalidate earlier experiments showing a chemotactic function of netrin-1, since DCC-dependent deposition suggests that netrin-1 may not be substrate-adhered at all times. These experiments together question whether netrin-1 functions as a chemotactic cue, and potentially suggest that netrin-1 may signal differently through chemotactic and haptotactic mechanisms, or perhaps that other modulators of netrin-1 guidance are critical *in situ*. Clearly further experiments are required to determine the role of chemotactic and haptotactic netrin-1 responses *in vitro* and *in vivo*, however, the data accumulated over the past 30 years suggest that both mechanisms are important during nervous system development.

## Netrin-1 Receptors and their Mechanisms

Netrin-1 acts through a repertoire of membrane-spanning receptors with extracellular domains that bind netrin and cytoplasmic domains that interact with effector proteins. These receptors include DCC (Frazzled in *Drosophila*, Unc-40 in *C. elegans*), its paralog neogenin in vertebrates, the UNC5 family (Unc5/Unc-5 in both *Drosophila* and *C. elegans*), and DSCAM (DSCAM in *Drosophila* and *C. elegans*).

### Deleted in Colorectal Cancer (DCC)

Deleted in colorectal cancer is a transmembrane receptor of the immunoglobulin superfamily highly expressed in spinal commissural neurons (Keino-Masu et al., 1996), the retina (Gad et al., 2000; Johansson et al., 2001), and many projection neurons of the fore- and midbrain during embryonic development (Shu et al., 2000). DCC functions as a receptor for netrin-1 in both growth cone attraction and repulsion (Hamelin et al., 1993; Chan et al., 1996; Keino-Masu et al., 1996; Kolodziej et al., 1996; Fazeli et al., 1997; Colavita and Culotti, 1998; Finci et al., 2014). The extracellular portion of DCC consists of four Ig-like N-terminal domains followed by six fibronectin type-III (FN3) repeats (Keino-Masu et al., 1996). Structural studies reveal that netrin-1 binds in the area of the fifth and sixth FN3 domains, and that attractive axon guidance requires binding to two of these sites (Geisbrecht et al., 2003; Mille et al., 2009; Finci et al., 2014). The binding of a single molecule of netrin-1 to two receptors induces DCC homodimerization (Finci et al., 2014). As there are at least three binding sites on DCC for netrin-1, and three binding sites on netrin-1 for DCC (Finci et al., 2014, 2015; Xu et al., 2014), netrin-1 may link dimers to produce the larger-order clustering of DCC observed *in vitro* (Matsumoto and Nagashima, 2010; Xu et al., 2014; Gopal et al., 2016; Plooster et al., 2017). A similar clustering process increases adhesion avidity in the integrin/laminin receptor/ligand system (Carman and Springer, 2003; Iwamoto and Calderwood, 2015), and could represent a mechanism for the regulation of mechanical forces on the netrin-1/DCC complex.

**FIGURE 5:**
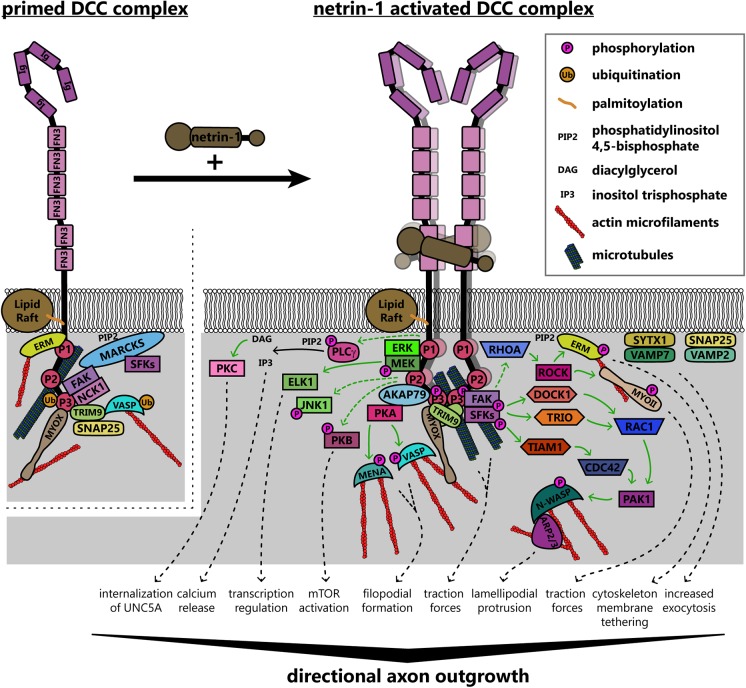
Model of known signaling pathways and interactions downstream of DCC in netrin-1 dependent axon guidance. DCC interacts with several enzymes and adaptor proteins and components of the actin and microtubule cytoskeletons in the absence of netrin-1, forming a “primed” signaling complex, which can rapidly initiate responses to ligand binding. Netrin-1 increases valency through multimerization of DCC homodimers. This clustering brings intracellular domains of the receptors into close apposition, forming a scaffold for the recruitment and activation of several proteins. Solid green arrows denote direct steps in activation, whereas dashed green arrows are known connections that may have intermediates. These pathways together modify the intracellular environment to promote directional axon outgrowth in response to netrin-1. AKAP79, A kinase anchoring protein 79; ARP2/3, actin-related protein 2/3 complex; CDC42, cell division control protein 42; DCC, deleted in colorectal cancer; DOCK1, dedicator of cytokinesis 1; ELK1, ETS transcription factor; ERK, mitogen activated protein kinase; ERM, ezrin-radixin-moesin; FAK, focal adhesion kinase; FN3, fibronectin type 3 domain; Ig, immunoglobulin domain; JNK1, c-Jun N-terminal kinase 1; MARCKS, myristoylated alanine-rich C kinase substrate; MEK, mitogen activated protein kinase kinase; MENA, mammalian enabled; MYOII, myosin II; MYOX, unconventional myosin X; NCK1, NCK adaptor protein 1; N-WASP, neuronal Wiskott–Aldrich syndrome protein; PAK1, protein associated kinase 1; PKA, protein kinase A; PKB, protein kinase B; PKC, protein kinase C; PLCγ, phospholipase C gamma; RAC1, Ras-related C3 botulinum toxin substrate 1; RHOA, Ras homolog gene family member A; ROCK, Rho associated protein kinase; SFKs, Src family kinases; SNAP25, synaptosomal associated protein 25; SYTX1, syntaxin-1; TIAM1, T-lymphoma invasion and metastasis protein 1; TRIM9, tripartite motif protein 9; TRIO, triple domain functional protein; VAMP2, vesicle associated membrane protein 2; VAMP7, vesicle associated membrane protein 7; VASP, vasodilator stimulated phosphoprotein.

The increase in receptor valence due to netrin-1:DCC multimerization likely brings the intracellular domains of the receptors into close apposition (Finci et al., 2014). The intracellular region of DCC contains three domains termed P1, P2, and P3, which are conserved among DCC family proteins (Kolodziej et al., 1996). When multiple DCC molecules coalesce due to netrin-1-dependent clustering, the P3 domains interact (Stein et al., 2001), and these binding-site-rich intracellular domains form a scaffold for the recruitment of downstream effectors and regulatory proteins (Li et al., 2002a). As the intracellular domains of DCC are required for repulsive netrin-1 dependent axon responses as well as attraction, parsing discrete downstream signaling pathways is complicated. This section specifically addresses mechanisms associated with either attractive axon guidance or increased axon outgrowth in response to netrin-1 downstream of DCC. Asymmetrical changes in the shape and rate of extension of the growth cone reorient outgrowth during turning; this involves dramatic and regulated remodeling of the plasma membrane (Quiroga et al., 2017) and underlying cytoskeleton (Dent et al., 2011), which is orchestrated by both chemical and mechanical transduction downstream of netrin/DCC, summarized in **Figure 5**.

#### DCC Interactions With the Cytoskeleton

The P3 domain of DCC is a hotspot for interaction with many binding partners of DCC that are poised to promote cytoskeletal and membrane remodeling, including the unconventional myosin X (MyoX), the non-receptor tyrosine kinase FAK, the E3 ubiquitin ligase TRIM9, F-actin binding ezrin-radixin-moesin (ERM) proteins, and p120RasGAP, which are situated to modulate chemical signaling, mechanotransduction, or both. The MyTH4-FERM domain of MyoX binds to the P3 domain of DCC and to microtubules, whereas the head/motor domain of MyoX translocates along filamentous actin; as such, MyoX translocates DCC to the periphery of cells and tips of filopodia (Zhu et al., 2007; Wei et al., 2011). MyoX is also required for netrin-1 dependent axon outgrowth and guidance of spinal commissural neurons (Zhu et al., 2007). Now that conditional alleles for MyoX exist (Heimsath et al., 2017), exploring the roles of MyoX in netrin dependent mechanotransduction or the formation of specific axon tracts in the brain is possible. DCC also reciprocally regulates MyoX, increasing association with actin filaments and promoting filopodial formation (Liu et al., 2012). The modulation of MyoX localization, and potentially function, by DCC represents an intriguing direction for future studies into the effect of extracellular ligands on intracellular force generation, however, future studies need to confirm the netrin dependency of MyoX enhanced actin binding.

Netrin-dependent remodeling of the actin and microtubule cytoskeletons are critical points in axon guidance that may also be regulated by mechanotransduction. The formation of filopodia in netrin-1 dependent axon guidance relies on the Ena/VASP family of actin polymerases (Gitai et al., 2003; Lebrand et al., 2004) which, along with DCC, localize to the tips of growth cone filopodia (Lanier et al., 1999; Shekarabi and Kennedy, 2002; Applewhite et al., 2007; Gupton and Gertler, 2007; Menon et al., 2015). Increases in the length and number of filopodia involves PKA phosphorylation of Mena at S236, corresponding to S157 in VASP (Lebrand et al., 2004; Deming et al., 2015). VASP, but not Mena or the third Ena/VASP family member EVL, is also regulated in netrin-1 dependent axon guidance by non-degradative TRIM9-dependent ubiquitination (Menon et al., 2015). Ubiquitination of VASP is associated with reduced filopodia lifetime and reduced VASP dynamics and localization to filopodia tips. The PKA-dependent phosphorylation of Ena/VASP proteins downstream of DCC also requires the function of PKA anchoring protein 79 (AKAP79) and ERM actin-binding proteins (Martín et al., 2006; Deming et al., 2015). In addition to regulation of actin-binding proteins, involvement of the microtubule cytoskeleton is also implicated in netrin-1/DCC responses: a recent study found that DCC interacted with neuron-specific β-III-tubulin in a netrin-1 dependent manner (Huang et al., 2018). By mutating sites on β-III-tubulin necessary for this binding, the group found that DCC:β-III-tubulin interactions are required for axon branching of cortical neurons and guidance of spinal commissural neurons.

#### DCC Signaling Pathways: Receptor-Linked Kinases and GEFs

Netrin-dependent dimerization of the DCC P3 domains results in clustering of FAK and adaptor protein NCK1, which are constitutively bound to DCC (Li et al., 2002a, 2004; Ren et al., 2004; Xu et al., 2018). This leads to activation of FAK through autophosphorylation at Y397, subsequent recruitment of Src family kinases (SFK), notably Fyn, which then phosphorylate FAK at Y861 and DCC at Y1418 (Li et al., 2004; Meriane et al., 2004; Ren et al., 2004). The autophosphorylation and activation of FAK also requires binding to phosphatidylinositol 4,5-bisphosphate [PI(4,5)P_2_, PIP2] at the membrane (Zhou et al., 2015). Intriguingly, FAK and SFK are maintained in a ready pool when not phosphorylated by the membrane-associated scaffolding protein myristoylated alanine-rich C kinase substrate (MARCKS) (Brudvig et al., 2018), which interacts with PIP2 and β-actin and promotes lamellipodium formation (Yamaguchi et al., 2009). This provides another link between the actin cytoskeleton and the membrane to transduce internal mechanical forces to the cell surface for remodeling, through the interaction between FAK and actin.

**FIGURE 6:**
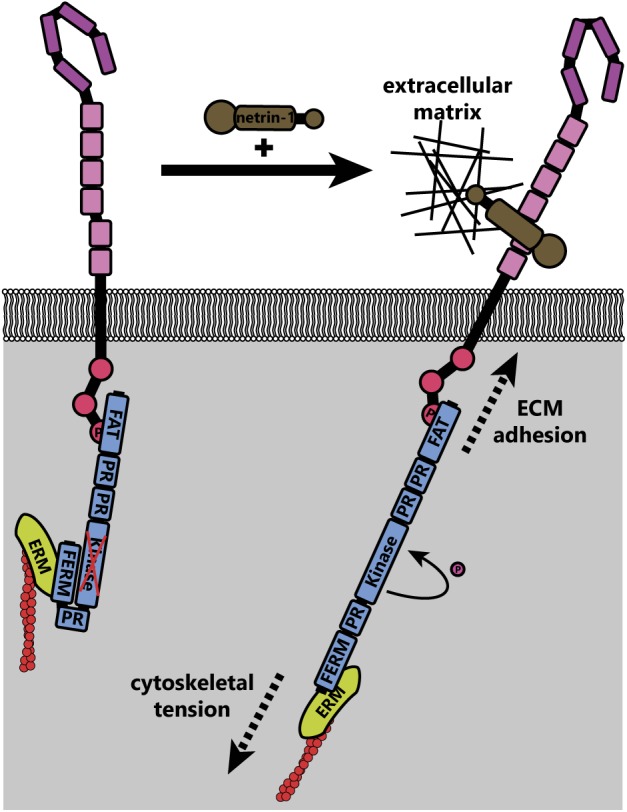
Theoretical model of FAK activation by mechanical forces (adapted from Moore et al., 2012). In the inactive state, the N-terminal FERM domain of FAK interacts with the kinase domain, preventing activation. Upon netrin-1 binding, tension on the actin cytoskeleton (through actin treadmilling and motor protein function) is transduced through ezrin-radixin-moesin (ERM) proteins to the N-terminus of FAK. The immobilization of the C-terminus of FAK through interaction with DCC, which is adhered to the extracellular matrix through its interaction with netrin-1, leads to increased mechanical force across FAK. This causes a conformational change that exposes the kinase domain, allowing autophosphorylation and subsequent activation of FAK.

Activation of FAK downstream of netrin-1 may be mechanically induced (Moore et al., 2012). The C-terminal FAT domain of FAK binds to the P3 domain of DCC, whereas the extracellular region of DCC is linked to the extracellular matrix through netrin-1 (**Figure 6**); FAK also indirectly interacts with the actin cytoskeleton through associations between its N-terminal FERM domain and the F-actin binding ERM proteins and Arp2/3 complex (Poullet et al., 2001; Serrels et al., 2007). These interactions together form a mechanotransductive complex centered on FAK. Forces generated by the treadmilling of connected actin filaments or contractile forces generated by myosins, expose the FAK kinase domain and allow autophosphorylation of FAK (Cooper et al., 2003). Further experiments are needed to identify the source of the mechanical forces that activate FAK upon netrin-1 binding to DCC. Additionally, atomic force microscopy could be conducted on netrin-1/DCC/FAK/actin complexes or FAK under tension *in vitro*, or models made *in silico* to determine the structure of FAK under tension and in a relaxed state.

Similar studies are needed to determine the extent to which mechanical forces are required for the activation of other proteins linking DCC to cytoskeletal components. For example netrin-dependent phosphorylation of DCC pY1418 recruits members of the ERM family (Martín et al., 2006; Antoine-Bertrand et al., 2011), linking DCC to the actin cytoskeleton. ERM proteins bridge filamentous actin to the plasma membrane through interaction with the transmembrane receptors CD44 (Mori et al., 2008) and L1 cell adhesion molecule (L1CAM) (Dickson et al., 2002), each of which have roles in nervous system development (Schmid and Maness, 2008; Raber et al., 2014). The ERM family member moesin also interacts with microtubules (Solinet et al., 2013), providing an additional link between the netrin-1/DCC complex, the membrane and the cytoskeleton that could transduce mechanical forces either into the cell for activation of signaling, or to the cell surface to generate remodeling and movement of the growth cone. Inhibition of ERM proteins in rat cortical neurons inhibits netrin-mediated axon outgrowth (Antoine-Bertrand et al., 2011). Activation of ERM (Mackay et al., 1997; Bretscher et al., 2002; Fehon et al., 2010; Ponuwei, 2016) and MYOII (Brown and Bridgman, 2003; Aguilar-Cuenca et al., 2014) enacts reorganization and traction forces, respectively, on the actin cytoskeleton.

Once activated by FAK, the SFKs Src and Fyn recruit to the DCC signaling complex the Rho GEFs DOCK180 (DOCK1) (Li et al., 2008) and triple domain functional protein (Trio) (Bateman et al., 2000; Forsthoefel et al., 2005; Briancon-Marjollet et al., 2008; DeGeer et al., 2013, 2015; Norris et al., 2014) to promote the activation of the Rho GTPase Rac1. Similarly the GEF Tiam-1 is recruited (Li et al., 2002b; Shekarabi and Kennedy, 2002; Shekarabi et al., 2005; Demarco et al., 2012) to activate Cdc42. In contrast, RhoA activity is decreased throughout the cell after application of netrin-1 in rat spinal commissural neurons (Moore et al., 2008). Interestingly, another study shows that netrin-1 treatment can also activate RhoA downstream of DCC in rat cortical neurons, leading to activation of Rho kinase (ROCK) and subsequent phosphorylation of and activation of ERM proteins (Antoine-Bertrand et al., 2011) and myosin II (MYOII) (Murray et al., 2010). Together these GTPases regulate the actin cytoskeleton to promote netrin-dependent spreading of lamellipodia and formation of filopodia (Moore et al., 2008). Activated Rac1 and Cdc42 form a complex with and activate p21-activated kinase 1 (PAK1), leading to the activation of the nucleation promotion factor, neuronal Wiskott–Aldrich syndrome protein (N-WASP) (Shekarabi et al., 2005). N-WASP then activates the filamentous actin nucleation and branching activity of the Arp2/3 complex, a major driver of lamellipodial protrusions (Mullins, 2000; Pollard et al., 2000). This pathway appears to be evolutionarily conserved, as the Rac-like GTPases CED-10 and MIG-2, and the GEF UNC-73 (ortholog of mammalian Trio) in *C. elegans* are also required for unc-6 dependent axon guidance (Gitai et al., 2003; Norris et al., 2014). However, mammalian Trio is required for attractive axon guidance, whereas the *C. elegans* ortholog is involved in repellant guidance (Bateman et al., 2000; Gitai et al., 2003). While GEF/GTPase activation is one of the most thoroughly studied components of netrin-1:DCC signaling, there remain unanswered questions to be investigated. For example, how do we reconcile the decrease in RhoA activity seen globally (Moore et al., 2008) with the observation that RhoA is activated downstream of DCC (Antoine-Bertrand et al., 2011)? Two potential explanations are that the activation and inhibition of specific GTPases are regulated differently depending upon timing after netrin-1 treatment, or that other signals dictate the direction of regulation. More work must be done to understand the intricacies of regulatory pathways and timelines in the context of DCC, as well as axon guidance receptors in general.

Deleted in colorectal cancer is also involved in activating members of the mitogen-activated protein kinase (MAPK) pathway (Campbell and Holt, 2003). Netrin-1 promotes recruitment, interaction, and activation of MAP kinase kinase 1 (MEK1), extracellular signal-related kinases 1 and 2 (ERK1/2), and Jun N-terminal kinase (JNK1) with DCC (Forcet et al., 2002; Ma et al., 2010; Qu et al., 2013). This is specific, as activation of another MAPK family member, p38 is not triggered by netrin-1 (Forcet et al., 2002). Palmitoylation of the transmembrane domain of DCC, and its association with lipid rafts is required for the activation of ERK (Hérincs et al., 2005). Activation of ERK is necessary for attractive axon guidance and leads to activation of transcription factors such as ELK1, suggesting a possible role of netrin-1/DCC signaling in transcriptional regulation (Forcet et al., 2002). DCC pY1418 is also a binding site for p120RasGAP, which is required to tightly control Ras and ERK activities in neurons during attraction of cortical axons toward netrin-1 (Antoine-Bertrand et al., 2016).

#### DCC Signaling Pathways: Plasma Membrane Remodeling

Recent work has elaborated on an important role of membrane reorganization and addition during netrin dependent axonal morphogenesis, along with regulation of membrane composition. Several groups described addition of DCC to the plasma membrane through exocytosis in response to netrin-1 (Bouchard et al., 2004, 2008; Matsumoto and Nagashima, 2010). The delivery of membrane is critical for axon guidance and outgrowth, which involve rapid plasma membrane expansion (Tojima et al., 2007, 2014; Urbina et al., 2018). Exocytosis frequency increases in response to netrin-1 (Winkle et al., 2014), and may occur downstream of DCC, FAK (Gupton and Gertler, 2007; Plooster et al., 2017), SFKs, and ERK1/2 (Ros et al., 2015). Early reports suggested that syntaxin-1 and vesicle associated membrane protein 7 (VAMP7, TI-VAMP) were required for this DCC-dependent increase in exocytosis in spinal commissural neurons, whereas synaptosomal associated protein 25 (SNAP25) and vesicle associated membrane protein 2 (VAMP2) were not involved (Cotrufo et al., 2011, 2012). However, later reports show that SNAP25 is required for SNARE complex formation and exocytosis in netrin-1 dependent axon branching in embryonic cortical neurons, and that SNAP25 is regulated in this context by TRIM9 (Winkle et al., 2014; Urbina et al., 2018). Studies also show an increase in the exocytosis frequency of VAMP2 containing vesicles in response to netrin-1 which, intriguingly, were regulated by TRIM9 specifically in neurites (Plooster et al., 2017; Urbina et al., 2018). Tension on the cell membrane has been shown to increase the efficiency of exocytosis (Kliesch et al., 2017); this membrane tension can be generated by increased cytoskeletal dynamics (Sens and Plastino, 2015), which are induced by netrin-1 activation of the signaling pathways detailed above. The increase in exocytosis in response to netrin-1 provides another avenue to investigate the role of intracellular force generation on axon guidance.

The plasma membrane lipid composition is also modified by netrin-1 signaling. Downstream of DCC, phospholipase Cγ (PLCγ) is phosphorylated and activated; this activation is required for axon outgrowth in response to netrin-1 (Ming et al., 1999; Xie et al., 2006). Activation of PLCγ leads to hydrolysis of PI(4,5)P_2_ into diacylglycerol (DAG) and inositol 1,4,5-trisphosphate, which among many other functions, lead to the activation of PKC and induce endoplasmic reticulum Ca^2+^ release, respectively (Patterson et al., 2005; Kadamur and Ross, 2013). As mentioned, PKC activation induces endocytosis of UNC5, which may promote increased DCC-only homodimerization and thus attractive outgrowth (Williams et al., 2003; Bartoe et al., 2006). Intracellular Ca^2+^ release is also required for netrin-1 dependent attraction (Hong et al., 2000). Interestingly, since PI(4,5)P_2_ is also required for autophosphorylation and activation of FAK by DCC (Zhou et al., 2015), this may represent an intrinsic homeostatic negative feedback loop that decays the DCC signal, giving it a finite lifetime. In addition to phosphatidylinositol hydrolysis, netrin-1 treatment also induces activation of PI3Ks in a DCC-dependent manner, and subsequent activation of protein kinase B (PKB/Akt) (Ming et al., 1999; Xie et al., 2006), which is necessary for nervous system development (Yang et al., 2004).

### Neogenin

Neogenin is well characterized as a receptor for the repulsive guidance molecule (RGM) (De Vries and Cooper, 2008). In contrast, although neogenin also binds netrin-1, most information regarding the function of neogenin in netrin-1 dependent axon guidance derives from comparisons made to DCC, as they are similar proteins with identical domain structures. In the spinal cord of the chicken, an organism in which there is no *DCC*, neogenin may fully replace DCC function (Phan et al., 2011). However, whether this comparison is valid in species with both paralogs remains to be seen. Some differences in protein functions have been noted. For example, whereas DCC promotes MyoX movement along basal actin filaments and induces basal filopodia formation, neogenin promotes MyoX movement toward apical filaments, and induces apical filopodia formation (Zhu et al., 2007; Wei et al., 2011). Further studies into the cause of this distinct regulation of MyoX may provide insight into the regulation of intracellular force generation by axon guidance receptors. DCC and neogenin may share common downstream signaling components, as neogenin interacts with DOCK1 as DCC does (Li et al., 2008). Unlike DCC, however, neogenin does not promote PI(4,5)P_2_ hydrolysis in response to netrin-1 (Xie et al., 2006), and therefore does not recruit an identical repertoire of proteins. Neogenin appears to facilitate spinal commissure formation alongside DCC in mice (Xu et al., 2014), as well as ventral forebrain axon tracts in *Xenopus* (Wilson and Key, 2006). Expression studies of neogenin in the developing mouse show a broad expression among many types of maturing neurons, suggesting the receptor may act in a variety of processes in addition to netrin-1 axon guidance (Fitzgerald et al., 2006). Future studies are needed to address the functional outcome of netrin-1 binding to neogenin, as this likely forms an isolated dimer as opposed to the clustering observed with DCC (Xu et al., 2014). As neogenin can functionally replace DCC in the chicken spinal cord, any differences between the signaling pathways and force transduction capacities of these two proteins could provide invaluable insight into which mechanisms are necessary for the formation of a spinal commissure.

### The UNC5 Family

**FIGURE 7:**
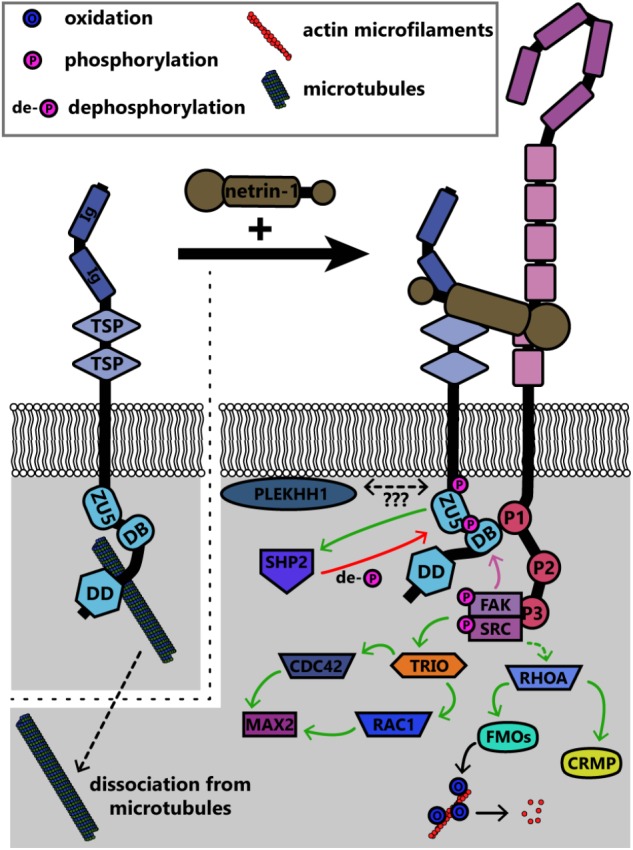
Summary of known signaling pathways downstream of UNC5 in repulsive netrin-1 signaling. Interaction between the intracellular DB domain of UNC5 and the P1 domain of DCC produces a scaffold similar to that in DCC homodimers. FAK and Src are phosphorylated and activated as in attractive netrin-1 signaling, however, the functional outcomes are different. CDC42, cell division control protein 42; CRMP, collapsin response mediating protein; DB, DCC-binding domain; DD, death domain; FAK, focal adhesion kinase; FMOs, Flavin monooxygenases; Ig, immunoglobulin domain; MAX2, more axillary growth 2; PLEKHH1, pleckstrin homology MyTH and FERM domain containing protein H1; RAC1, Ras-related C3 botulinum toxin substrate 1; RHOA, Ras homolog gene family member A; SHP2, protein tyrosine phosphatase 2C; SRC, proto-oncogene tyrosine-protein kinase Src; TRIO, triple domain functional protein; TSP, thrombospondin type 1 domain; ZU5, ZO-1/Unc5 domain.

The UNC5 family of repulsive netrin receptors consists of one isoform in invertebrates (UNC5 in *Drosophila* and Unc-5 in *C. elegans*) and up to four in vertebrates (UNC5A, UNC5B, UNC5C, UNC5D). UNC5 proteins function in repulsive axon guidance in response to netrin-1, through heterodimerization with DCC for long-range repulsion (Hamelin et al., 1993; Colavita and Culotti, 1998; Finci et al., 2014), and potentially heterodimerization with DSCAM for short-range repulsion (Keleman and Dickson, 2001; Purohit et al., 2012). The signaling pathways discussed in this section are summarized in **Figure 7**. The intracellular domain of UNC5 contains a binding site for the P1 domain of DCC; association between UNC5 and DCC mediates long-range netrin-1 dependent axon repulsion (Hong et al., 1999; Finci et al., 2014). DCC independent repulsion effects also require the ZU-5 motif of the UNC5 intracellular domain (Killeen et al., 2002) and the adaptor protein required for motor axon guidance 1 (Max-1, human homolog PLEKHH1) (Huang et al., 2002), both of which may scaffold downstream proteins into a signaling complex. The heterodimer of DCC and UNC5 recruits many of the same effectors for repulsion as DCC homodimers recruit for attractive signaling. Notably, Src and FAK are recruited by DCC/UNC5 heterodimers upon netrin-1 stimulation, leading to phosphorylation of *C. elegans* Unc-5 at Y482 (Y568 in murine UNC5C) (Tong et al., 2001). This phosphorylation of Unc-5 is necessary for repulsive netrin-1 axon guidance *in vivo* (Killeen et al., 2002; Li et al., 2006). Phosphorylation of UNC5C recruits tyrosine-protein phosphatase non-receptor type 11 (PTPN11/SHP2), which then dephosphorylates UNC5C (Tong et al., 2001). Thus, phosphorylation may lead to activation of proteins downstream of UNC5, but additionally activates negative feedback through phosphatases like SHP2 as possible homeostatic mechanisms.

**FIGURE 8:**
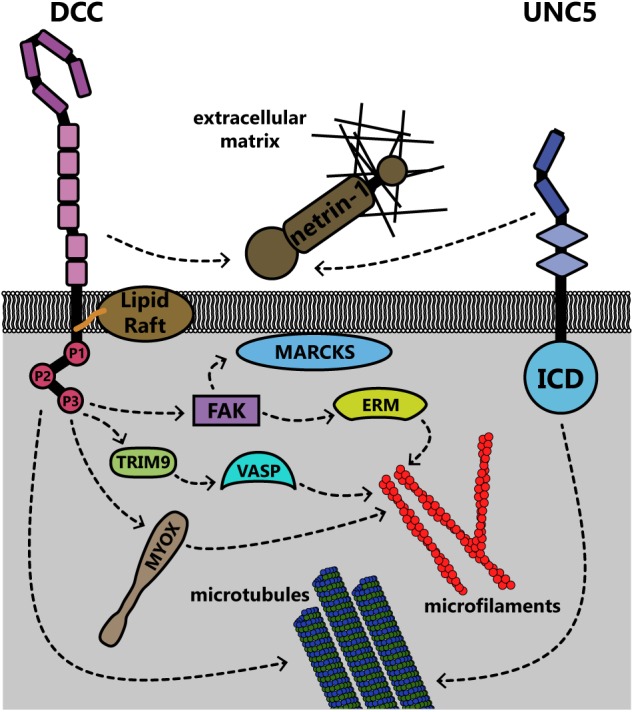
Summary of known interactions linking the transmembrane receptors DCC and UNC5 to the membrane and cytoskeleton. Both receptors are connected to the extracellular matrix through interaction with netrin-1. DCC, deleted in colorectal cancer; ERM, ezrin-radixin-moesin; ICD, intracellular domain; MARCKS, myristoylated alanine-rich C kinase substrate; MYOX, unconventional myosin X; TRIM9, tripartite motif protein 9; UNC5, uncoordinated locomotion 5; VASP, vasodilator stimulated phosphoprotein.

Rho family GTPases are also regulated downstream of Unc5 through distinct repertoires of GEFs. In neurite outgrowth in mouse neuroblastoma cells, RhoA is activated by netrin-1 binding to UNC5A, and to a lesser extent, Rac1 and Cdc42 are activated (Picard et al., 2009). In *C. elegans*, netrin-1 binding to Unc-5 similarly leads to activation of the Trio ortholog Unc-73, which in repulsive netrin-1 responses can act as a GEF for the GTPases Ced-10 and Mig-2 (as opposed to Tiam-1, which acts as a GEF for these GTPases in attractive netrin-1 responses [Li et al., 2002b; Demarco et al., 2012) to inhibit growth cone protrusion (Norris et al., 2014)]. Downstream of these GTPases, the PAK Max-2 is required for netrin-1 dependent repulsion (Lucanic et al., 2006). Recent work demonstrates that additional effectors of these GTPases in netrin-1 repulsion include Unc-33 [ortholog of collapsin response mediator proteins (CRMPs)], which regulates remodeling of both the actin and microtubule cytoskeletons (Quach et al., 2015), and Unc-44 (ortholog of Ankyrin), an adaptor protein connecting integral membrane proteins to the spectrin cytoskeleton (Rubtsov and Lopina, 2000; Norris et al., 2014). Genetic analysis suggests the flavin monooxygenases Fmo-1, Fmo-4, Fmo-5 and Ebhp-1 are required for netrin-dependent repulsion in *C. elegans* (Gujar et al., 2017). The *C. elegans* Rac orthologs activate the flavin monooxygenases, which oxidize a variety of substrates (Huijbers et al., 2014). A similar family of monooxygenases, the molecule interacting with CasL (MICAL) protein family, has been shown in *Drosophila* and in vertebrates to regulate repulsive axon guidance to semaphorins by oxidative dismantling of F-actin (Terman et al., 2002; Hung et al., 2010; Grintsevich et al., 2016). However, whether the *C. elegans* monooxygenases similarly oxidize F-actin or other relevant substrates in netrin-1 dependent axon guidance has yet to be investigated. In addition to regulation of the actin cytoskeleton, UNC5C also interacts with β-III-tubulin, an interaction that may modulate netrin-1 dependent axon repulsion (Shao et al., 2017). This represents yet another link between the transmembrane netrin-1 receptors and the cytoskeleton; all of these links are summarized for DCC and UNC5 in **Figure 8**.

### Down Syndrome Cell Adhesion Molecule (DSCAM)

Down syndrome cell adhesion molecule is a transmembrane cell adhesion molecule that also acts as a receptor for netrin-1 (Agarwala et al., 2001; Hattori et al., 2008; Montesinos, 2014). DSCAM acts with UNC5 as a co-receptor for netrin-1, to mediate short-range repulsive responses (Purohit et al., 2012). DSCAM is required for midline crossing of spinal commissural neurons in *Xenopus*, and neurons exogenously expressing DSCAM respond to attractive netrin-1 independent of DCC (Ly et al., 2008). However, DSCAM is not required for all netrin-1 mediated guidance, such as in mouse spinal commissural neurons (Palmesino et al., 2012). In *Drosophila*, DSCAM functions as a midline receptor for netrin and possibly for an unidentified guidance cue operating in parallel to netrin and the DCC ortholog Frazzled (Andrews et al., 2008). DSCAM interacts with UNC5C in response to netrin-1, and this pair mediates axonal growth cone collapse in mouse cerebellar granule cells by promoting the phosphorylation of FAK, Fyn, and PAK1 (Liu et al., 2009; Purohit et al., 2012). Evidence also suggests that DSCAM may coordinate with DCC to regulate interactions with microtubules (Huang et al., 2015) and the activation of JNK1 in response to netrin-1 (Qu et al., 2013). Possibly independently of either UNC5 or DCC, DSCAM interacts with AMP-activated protein kinase (AMPK), which is phosphorylated in response to netrin-1 treatment (Zhu et al., 2013). DSCAM promotes axonal fasciculation and dendritic tiling through neurite-neurite adhesion (Millard and Zipursky, 2008; Tadros et al., 2016; Cohen et al., 2017). Further studies are required to mechanistically define how DSCAM mediates diverse functions during netrin-1 dependent morphogenesis.

## New Netrin-1 Modifiers

**FIGURE 9:**
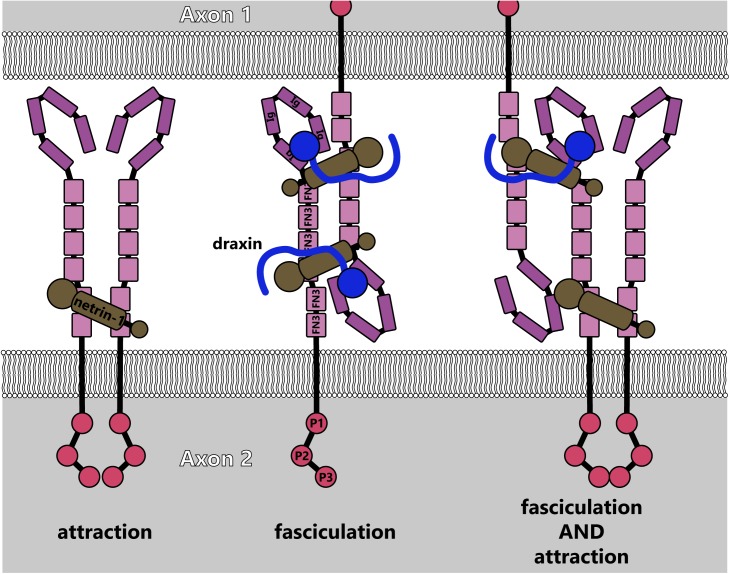
Model for fasciculation induced by draxin-netrin-1 interaction with DCC (adapted from Liu et al., 2018). Interaction between draxin and netrin-1 potentially links together DCC receptors from adjacent axons, causing fasciculation. This could additionally lead to higher-order multimers of DCC, which would induce attractive signaling cascades along with linking axons.

Recently identified netrin-1 modifiers provide additional complexity to netrin-dependent axonal morphogenesis. The protein draxin, secreted from the floor plate, acts as an antagonist in the developing spinal cord by binding to netrin (Gao et al., 2015). Structural studies demonstrate that a draxin/netrin-1 complex binds two DCC molecules from neighboring axons (in *trans*) simultaneously, promoting fasciculation as opposed to attractive axon guidance or outgrowth (Liu et al., 2018), as illustrated in **Figure 9**. In light of recent studies that revealed floor-plate-derived netrin-1 is dispensable for commissural axon guidance (Dominici et al., 2017; Varadarajan et al., 2017; Yamauchi et al., 2017), a mechanism in which soluble netrin-1 is converted to a fasciculation signal at the location of commissure formation may be an exciting avenue for future study. Studies on draxin in the central nervous system suggest a similar role there as well. Deletion of draxin in mice leads to partially penetrant agenesis of the corpus callosum, a major midline brain commissure (Ahmed et al., 2011). Concomitant heterozygous deletion of *Dcc* and *draxin* (*Dcc*^+/-^*draxin*^+/-^) results in a much stronger agenesis phenotype than either single heterozygous knockout.

Structural studies suggest that netrin-1 may require heparin sulfates as co-ligands, as the C-terminal extracellular matrix domain of netrin-1 interacts with GAGs (Kappler et al., 2000; Geisbrecht et al., 2003), and X-ray crystallography of netrin-1 in complex with DCC reveals a bound sulfate (Finci et al., 2014). A recent study also shows that the heparin sulfate proteoglycan (HSPG) glypican binds to DCC and modulates netrin-1 dependent axon guidance *in vivo* (Blanchette et al., 2015). Curiously, this interaction with netrin-1/DCC relies on the N-terminal peptide-only region of glypican rather than on its C-terminal heparin sulfate binding domain. However, the complex structure of a proteoglycan such as glypican, which is membrane-bound by its C-terminus, could allow for mechanical attachments between netrin-1, DCC, cell membranes and the extracellular matrix. Further studies are needed to assess the extent of proteoglycan regulation of netrin-1 signaling pathways, and the mechanisms by which glypican and heparin sulfates influence chemotactic and haptotactic axon guidance.

## Author Contributions

Both authors made substantial, direct, and intellectual contributions to the work, and approved it for publication.

## Conflict of Interest Statement

The authors declare that the research was conducted in the absence of any commercial or financial relationships that could be construed as a potential conflict of interest.

## References

[B1] Agarwala K. L. Ganesh S. Suzuki T. Akagi T. Kaneko K. Amano K. (2001). Dscam is associated with axonal and dendritic features of neuronal cells. *J. Neurosci. Res.* 66 337–346. 10.1002/jnr.1226 11746351

[B2] Aguilar-Cuenca R. Juanes-García A. Vicente-Manzanares M. (2014). Myosin II in mechanotransduction: master and commander of cell migration, morphogenesis, and cancer. *Cell. Mol. Life Sci.* 71 479–492. 10.1007/s00018-013-1439-5 23934154 PMC11113847

[B3] Ahmed G. Shinmyo Y. Ohta K. Islam S. M. Hossain M. Naser I. B. (2011). Draxin inhibits axonal outgrowth through the Netrin receptor DCC. *J. Neurosci.* 31 14018–14023. 10.1523/JNEUROSCI.0943-11.2011 21957262 PMC6633171

[B4] Andrews G. L. Tanglao S. Farmer W. T. Morin S. Brotman S. Berberoglu M. A. (2008). Dscam guides embryonic axons by Netrin-dependent and -independent functions. *Development* 135 3839–3848. 10.1242/dev.023739 18948420 PMC2712571

[B5] Antoine-Bertrand J. Duquette P. M. Alchini R. Kennedy T. E. Fournier A. E. Lamarche-Vane N. (2016). p120RasGAP protein mediates netrin-1 protein-induced cortical axon outgrowth and guidance. *J. Biol. Chem.* 291 4589–4602. 10.1074/jbc.M115.674846 26710849 PMC4813483

[B6] Antoine-Bertrand J. Ghogha A. Luangrath V. Bedford F. K. Lamarche-Vane N. (2011). The activation of ezrin-radixin-moesin proteins is regulated by netrin-1 through Src kinase and RhoA/Rho kinase activities and mediates netrin-1-induced axon outgrowth. *Mol. Biol. Cell* 22 3734–3746. 10.1091/mbc.E10-11-0917 21849478 PMC3183026

[B7] Applewhite D. A. Barzik M. Kojima S.-I. Svitkina T. M. Gertler F. B. Borisy G. G. (2007). Ena/VASP proteins have an anti-capping independent function in filopodia formation. *Mol. Biol. Cell* 18 2579–2591. 10.1091/mbc.E06-11-0990 17475772 PMC1924831

[B8] Bartoe J. L. McKenna W. L. Quan T. K. Stafford B. K. Moore J. A. Xia J. (2006). Protein interacting with c-kinase 1/protein kinase C -mediated endocytosis converts netrin-1-mediated repulsion to attraction. *J. Neurosci.* 26 3192–3205. 10.1523/JNEUROSCI.3469-05.2006 16554470 PMC6674106

[B9] Bateman J. Shu H. Van Vactor D. (2000). The guanine nucleotide exchange factor Trio mediates axonal development in the *Drosophila* embryo. *Neuron* 26 93–106. 10.1016/S0896-6273(00)81141-1 10798395

[B10] Benavides Damm T. Egli M. (2014). Calcium’s role in mechanotransduction during muscle development. *Cell. Physiol. Biochem.* 33 249–272. 10.1159/000356667 24525559

[B11] Bin J. M. Han D. Lai Wing Sun K. Croteau L.-P. Dumontier E. Cloutier J.-F. (2015). Complete loss of netrin-1 results in embryonic lethality and severe axon guidance defects without increased neural cell death. *Cell Rep.* 12 1099–1106. 10.1016/j.celrep.2015.07.028 26257176

[B12] Bixby J. L. Grunwald G. B. Bookman R. J. (1994). Ca^2+^ influx and neurite growth in response to purified N-cadherin and laminin. *J. Cell Biol.* 127 1461–1475. 10.1083/jcb.127.5.1461 7962102 PMC2120265

[B13] Blanchette C. R. Perrat P. N. Thackeray A. Bénard C. Y. (2015). Glypican is a modulator of netrin-mediated axon guidance. *PLoS Biol.* 13:e1002183. 10.1371/journal.pbio.1002183 26148345 PMC4493048

[B14] Bouchard J. F. Horn K. E. Stroh T. Kennedy T. E. (2008). Depolarization recruits DCC to the plasma membrane of embryonic cortical neurons and enhances axon extension in response to netrin-1. *J. Neurochem.* 107 398–417. 10.1111/j.1471-4159.2008.05609.x 18691385

[B15] Bouchard J.-F. Moore S. W. Tritsch N. X. Roux P. P. Shekarabi M. Barker P. A. (2004). Protein kinase A activation promotes plasma membrane insertion of DCC from an intracellular pool: a novel mechanism regulating commissural axon extension. *J. Neurosci.* 24 3040–3050. 10.1523/JNEUROSCI.4934-03.2004 15044543 PMC6729852

[B16] Brankatschk M. Dickson B. J. (2006). Netrins guide *Drosophila* commissural axons at short range. *Nat. Neurosci.* 9 188–194. 10.1038/nn1625 16429137

[B17] Bretscher A. Edwards K. Fehon R. G. (2002). ERM proteins and merlin: integrators at the cell cortex. *Nat. Rev. Mol. Cell Biol.* 3 586–599. 10.1038/nrm882 12154370

[B18] Briancon-Marjollet A. Ghogha A. Nawabi H. Triki I. Auziol C. Fromont S. (2008). Trio mediates netrin-1-induced rac1 activation in axon outgrowth and guidance. *Mol. Cell. Biol.* 28 2314–2323. 10.1128/MCB.00998-07 18212043 PMC2268419

[B19] Brown J. Bridgman P. C. (2003). Role of myosin II in axon outgrowth. *J. Histochem. Cytochem.* 51 421–428. 10.1177/002215540305100403 12642620

[B20] Brudvig J. J. Cain J. T. Schmidt-Grimminger G. G. Stumpo D. J. Roux K. J. Blackshear P. J. (2018). MARCKS Is necessary for Netrin-DCC signaling and corpus callosum formation. *Mol. Neurobiol.* 10.1007/s12035-018-0990-3 [Epub ahead of print]. 29546593 PMC6139093

[B21] Campbell D. S. Holt C. E. (2003). Apoptotic pathway and MAPKs differentially regulate chemotropic responses of retinal growth cones. *Neuron* 37 939–952. 10.1016/S0896-6273(03)00158-2 12670423

[B22] Carman C. V. Springer T. A. (2003). Integrin avidity regulation: Are changes in affinity and conformation underemphasized? *Curr. Opin. Cell Biol.* 15 547–556. 10.1016/j.ceb.2003.08.003 14519389

[B23] Ceresa B. (2012). Spatial regulation of epidermal growth factor receptor signaling by endocytosis. *Int. J. Mol. Sci.* 14 72–87. 10.3390/ijms14010072 23344022 PMC3565252

[B24] Chan S. S. Y. Zheng H. Su M. W. Wilk R. Killeen M. T. Hedgecock E. M. (1996). UNC-40, a *C. elegans* homolog of DCC (Deleted in Colorectal Cancer), is required in motile cells responding to UNC-6 netrin cues. *Cell* 87 187–195. 10.1016/S0092-8674(00)81337-9 8861903

[B25] Cohen O. Vald L. Yamagata M. Sanes J. R. Klar A. Ri I. (2017). Roles of DSCAM in axonal decussation and fasciculation of chick spinal interneurons. *Int. J. Dev. Biol.* 244 235–244. 10.1387/ijdb.160235ak 28621421

[B26] Colavita A. Culotti J. G. (1998). Suppressors of ectopic UNC-5 growth cone steering identify eight genes involved in axon guidance in *Caenorhabditis elegans*. *Dev. Biol.* 194 72–85. 10.1006/dbio.1997.8790 9473333

[B27] Cooper L. A. Shen T. Guan J.-L. (2003). Regulation of focal adhesion kinase by its amino-terminal domain through an autoinhibitory interaction regulation of focal adhesion kinase by its amino-terminal domain through an autoinhibitory interaction. *Mol. Cell. Biol.* 23 8030–8041. 10.1128/MCB.23.22.8030 14585964 PMC262338

[B28] Cotrufo T. Andrés R. M. Ros O. Pérez-Brangulí F. Muhaisen A. Fuschini G. (2012). Syntaxin 1 is required for DCC/Netrin-1-dependent chemoattraction of migrating neurons from the lower rhombic lip. *Eur. J. Neurosci.* 36 3152–3164. 10.1111/j.1460-9568.2012.08259.x 22946563

[B29] Cotrufo T. Perez-Branguli F. Muhaisen A. Ros O. Andres R. Baeriswyl T. (2011). A signaling mechanism coupling netrin-1/deleted in colorectal cancer chemoattraction to SNARE-mediated exocytosis in axonal growth cones. *J. Neurosci.* 31 14463–14480. 10.1523/JNEUROSCI.3018-11.2011 21994363 PMC6703395

[B30] De La Torre J. R. Höpker V. H. Ming G. L. Poo M. M. Tessier-Lavigne M. Hemmati-Brivanlou A. (1997). Turning of retinal growth cones in a netrin-1 gradient mediated by the netrin receptor DCC. *Neuron* 19 1211–1224. 10.1016/S0896-6273(00)80413-4 9427245

[B31] De Vries M. Cooper H. M. (2008). Emerging roles for neogenin and its ligands in CNS development. *J. Neurochem.* 106 1483–1492. 10.1111/j.1471-4159.2008.05485.x 18485097

[B32] DeGeer J. Boudeau J. Schmidt S. Bedford F. Lamarche-Vane N. Debant A. (2013). Tyrosine phosphorylation of the Rho guanine nucleotide exchange factor trio regulates Netrin-1/DCC-mediated cortical axon outgrowth. *Mol. Cell. Biol.* 33 739–751. 10.1128/MCB.01264-12 23230270 PMC3571336

[B33] DeGeer J. Kaplan A. Mattar P. Morabito M. Stochaj U. Kennedy T. E. (2015). Hsc70 chaperone activity underlies Trio GEF function in axon growth and guidance induced by netrin-1. *J. Cell Biol.* 210 817–832. 10.1083/jcb.201505084 26323693 PMC4555821

[B34] Deiner M. S. Kennedy T. E. Fazeli A. Serafini T. Tessier-lavigne M. Sretavan D. W. (1997). Netrin-1 and DCC mediate axon guidance locally at the optic disc: loss of function leads to optic nerve hypoplasia. *Neuron* 19 575–589. 9331350 10.1016/s0896-6273(00)80373-6

[B35] Demarco R. S. Struckhoff E. C. Lundquist E. A. (2012). The Rac GTP exchange factor TIAM-1 acts with CDC-42 and the guidance receptor UNC-40/DCC in neuronal protrusion and axon guidance. *PLoS Genet.* 8:e1002665. 10.1371/journal.pgen.1002665 22570618 PMC3343084

[B36] Deming P. B. Campbell S. L. Stone J. B. Rivard R. L. Mercier A. L. Howe A. K. (2015). Anchoring of protein kinase a by ERM (ezrin-radixin-moesin) proteins is required for proper netrin signaling through DCC (deleted in colorectal cancer). *J. Biol. Chem.* 290 5783–5796. 10.1074/jbc.M114.628644 25575591 PMC4342488

[B37] Dent E. W. Barnes A. M. Tang F. Kalil K. (2004). Netrin-1 and semaphorin 3A promote or inhibit cortical axon branching, respectively, by reorganization of the cytoskeleton. *J. Neurosci.* 24 3002–3012. 10.1523/JNEUROSCI.4963-03.2004 15044539 PMC6729836

[B38] Dent E. W. Gupton S. L. Gertler F. B. (2011). The growth cone cytoskeleton in axon outgrowth and guidance. *Cold Spring Harb. Perspect. Biol.* 3:a001800. 10.1101/cshperspect.a001800 21106647 PMC3039926

[B39] Dickson T. C. David Mintz C. Benson D. L. Salton S. R. J. (2002). Functional binding interaction identified between the axonal CAM L1 and members of the ERM family. *J. Cell Biol.* 157 1105–1112. 10.1083/jcb.200111076 12070130 PMC2173555

[B40] Dominici C. Moreno-Bravo J. A. Puiggros S. R. Rappeneau Q. Rama N. Vieugue P. (2017). Floor-plate-derived netrin-1 is dispensable for commissural axon guidance. *Nature* 545 350–354. 10.1038/nature22331 28445456 PMC5438598

[B41] Dun X. P. Parkinson D. B. (2017). Role of Netrin-1 signaling in nerve regeneration. *Int. J. Mol. Sci.* 18 1–22. 10.3390/ijms18030491 28245592 PMC5372507

[B42] Fazeli A. Dickinson S. L. Hermiston M. L. Tighe R. V. Steen R. G. Small C. G. (1997). Phenotype of mice lacking functional Deleted in colorectal cancer (Dcc) gene. *Nature* 386 796–804. 10.1038/386796a0 9126737

[B43] Fehon R. G. McClatchey A. I. Bretscher A. (2010). Organizing the cell cortex: the role of ERM proteins. *Nat. Rev. Mol. Cell Biol.* 11 276–287. 10.1038/nrm2866 20308985 PMC2871950

[B44] Finci L. Zhang Y. Meijers R. Wang J. (2015). Signaling mechanism of the netrin-1 receptor DCC in axon guidance. *Prog. Biophys. Mol. Biol.* 118 153–160. 10.1016/j.pbiomolbio.2015.04.001 25881791 PMC4537816

[B45] Finci L. I. Krüger N. Sun X. Zhang J. Chegkazi M. Wu Y. (2014). The crystal structure of netrin-1 in complex with DCC reveals the bi-functionality of netrin-1 as a guidance cue. *Neuron* 83 839–849. 10.1016/j.neuron.2014.07.010 25123307 PMC4412161

[B46] Fitzgerald D. P. Seaman C. Cooper H. M. (2006). Localization of neogenin protein during morphogenesis in the mouse embryo. *Dev. Dyn.* 235 1720–1725. 10.1002/dvdy.20744 16552762

[B47] Flores C. (2011). Role of netrin-1 in the organization and function of the mesocorticolimbic dopamine system. *J. Psychiatry Neurosci.* 36 296–310. 10.1503/jpn.100171 21481303 PMC3163646

[B48] Forcet C. Stein E. Pays L. Corset V. Llambi F. Tessier-Lavigne M. (2002). Netrin-1-mediated axon outgrowth requires deleted in colorectal cancer-dependent MAPK activation. *Nature* 417 443–447. 10.1038/nature748 11986622

[B49] Forsthoefel D. J. Liebl E. C. Kolodziej P. A. Seeger M. A. (2005). The Abelson tyrosine kinase, the Trio GEF and Enabled interact with the Netrin receptor Frazzled in Drosophila. *Development* 132 1983–1994. 10.1242/dev.01736 15790972

[B50] Gad J. M. Keeling S. L. Shu T. Richards L. J. Cooper H. M. (2000). The spatial and temporal expression patterns of netrin receptors, DCC and neogenin, in the developing mouse retina. *Exp. Eye Res.* 70 711–722. 10.1006/exer.2000.0823 10843775

[B51] Gao X. Metzger U. Panza P. Mahalwar P. Alsheimer S. Geiger H. (2015). A floor-plate extracellular protein-protein interaction screen identifies Draxin as a secreted Netrin-1 antagonist. *Cell Rep.* 12 694–708. 10.1016/j.celrep.2015.06.047 26190107

[B52] Geisbrecht B. V. Dowd K. A. Barfield R. W. Longo P. A. Leahy D. J. (2003). Netrin binds discrete subdomains of DCC and UNC5 and mediates interactions between DCC and heparin. *J. Biol. Chem.* 278 32561–32568. 10.1074/jbc.M302943200 12810718

[B53] Geng J. Zhao Q. Zhang T. Xiao B. (2017). “Chapter Six - In touch with the mechanosensitive Piezo channels: structure, ion permeation, and mechanotransduction,” in *Piezo Channels*, ed. Gottlieb P. (Cambridge, MA: Academic Press), 159–195. 10.1016/bs.ctm.2016.11.006 28728816

[B54] Gitai Z. Yu T. W. Lundquist E. A. Tessier-Lavigne M. Bargmann C. I. (2003). The netrin receptor UNC-40/DCC stimulates axon attraction and outgrowth through enabled and, in parallel, Rac and UNC-115/abLIM. *Neuron* 37 53–65. 10.1016/S0896-6273(02)01149-2 12526772

[B55] Glendining K. A. Markie D. Gardner R. J. M. Franz E. A. Robertson S. P. Jasoni C. L. (2017). A novel role for the DNA repair gene Rad51 in Netrin-1 signalling. *Sci. Rep.* 7:39823. 10.1038/srep39823 28057929 PMC5216413

[B56] Gopal A. A. Rappaz B. Rouger V. Martyn I. B. Dahlberg P. D. Meland R. J. (2016). Netrin-1-regulated distribution of UNC5B and DCC in live cells revealed by TICCS. *Biophys. J.* 110 623–634. 10.1016/j.bpj.2015.12.022 26840727 PMC4744167

[B57] Grintsevich E. E. Yesilyurt H. G. Rich S. K. Hung R.-J. Terman J. R. Reisler E. (2016). F-actin dismantling through a redox-driven synergy between Mical and cofilin. *Nat. Cell Biol.* 18 876–885. 10.1038/ncb3390 27454820 PMC4966907

[B58] Groc L. Choquet D. (2006). AMPA and NMDA glutamate receptor trafficking: multiple roads for reaching and leaving the synapse. *Cell Tissue Res.* 326 423–438. 10.1007/s00441-006-0254-9 16847641

[B59] Gujar M. R. Stricker A. M. Lundquist E. A. (2017). Flavin monooxygenases regulate *Caenorhabditis elegans* axon guidance and growth cone protrusion with UNC-6/Netrin signaling and Rac GTPases. *PLoS Genet.* 13:e1006998. 10.1371/journal.pgen.1006998 28859089 PMC5597259

[B60] Gupton S. L. Gertler F. B. (2007). Filopodia: the fingers that do the walking. *Sci. STKE* 2007:re5. 10.1126/stke.4002007re5 17712139

[B61] Hamelin M. Zhou Y. Su M.-W. Scott I. M. Culotti J. G. (1993). Expression of the UNC-5 guidance receptor in the touch neurons of *C. elegans* steers their axons dorsally. *Nature* 364 327–330. 8332188 10.1038/364327a0

[B62] Hattori D. Millard S. S. Wojtowicz W. M. Zipursky S. L. (2008). Dscam-mediated cell recognition regulates neural circuit formation. *Annu. Rev. Cell Dev. Biol.* 24 597–620. 10.1146/annurev.cellbio.24.110707.175250 18837673 PMC2711549

[B63] Hausott B. Klimaschewski L. (2016). Membrane turnover and receptor trafficking in regenerating axons. *Eur. J. Neurosci.* 43 309–317. 10.1111/ejn.13025 26222895

[B64] Hedgecock E. M. Culotti J. G. Hall D. H. (1990). The unc-5, unc-6, and unc-40 genes guide circumferential migrations of pioneer axons and mesodermal cells on the epidermis in *C. elegans*. *Neuron* 4 61–85. 10.1016/0896-6273(90)90444-K 2310575

[B65] Heimsath E. G. J. Yim Y. Mustapha M. Hammer J. A. Cheney R. E. (2017). Myosin-X knockout is semi-lethal and demonstrates that myosin-X functions in neural tube closure, pigmentation, hyaloid vasculature regression, and filopodia formation. *Sci. Rep.* 7:17354. 10.1038/s41598-017-17638-x 29229982 PMC5725431

[B66] Hérincs Z. Corset V. Cahuzac N. Furne C. Castellani V. Hueber A.-O. (2005). DCC association with lipid rafts is required for netrin-1-mediated axon guidance. *J. Cell Sci.* 118 1687–1692. 10.1242/jcs.02296 15811950

[B67] Hong K. Hinck L. Nishiyama M. Poo M. M. Tessier-Lavigne M. Stein E. (1999). A ligand-gated association between cytoplasmic domains of UNC5 and DCC family receptors converts netrin-induced growth cone attraction to repulsion. *Cell* 97 927–941. 10.1016/S0092-8674(00)80804-1 10399920

[B68] Hong K. Nishiyama M. Henley J. Tessier-Lavigne M. Poo M. (2000). Calcium signalling in the guidance of nerve growth by netrin-1. *Nature* 403 93–98. 10638760 10.1038/47507

[B69] Höpker V. H. Shewan D. Tessier-Lavigne M. Poo M. Holt C. (1999). Growth-cone attraction to netrin-1 is converted to repulsion by laminin-1. *Nature* 401 69–73. 10485706 10.1038/43441

[B70] Huang H. Shao Q. Qu C. Yang T. Dwyer T. Liu G. (2015). Coordinated interaction of Down syndrome cell adhesion molecule and deleted in colorectal cancer with dynamic TUBB3 mediates Netrin-1-induced axon branching. *Neuroscience* 293 109–122. 10.1016/j.neuroscience.2015.02.042 25754961 PMC4386621

[B71] Huang H. Yang T. Shao Q. Majumder T. Mell K. Liu G. (2018). Human TUBB3 mutations disrupt netrin attractive signaling. *Neuroscience* 374 155–171. 10.1016/j.neuroscience.2018.01.046 29382549 PMC5841466

[B72] Huang X. Cheng H. J. Tessier-Lavigne M. Jin Y. (2002). MAX-1, a novel PH/MyTH4/FERM domain cytoplasmic protein implicated in netrin-mediated axon repulsion. *Neuron* 34 563–576. 10.1016/S0896-6273(02)00672-4 12062040

[B73] Huijbers M. M. E. Montersino S. Westphal A. H. Tischler D. Van Berkel W. J. H. (2014). Flavin dependent monooxygenases. *Arch. Biochem. Biophys.* 544 2–17. 10.1016/j.abb.2013.12.005 24361254

[B74] Hung R.-J. Yazdani U. Yoon J. Wu H. Yang T. Gupta N. (2010). Mical links semaphorins to F-actin disassembly. *Nature* 463 823–827. 10.1038/nature08724 20148037 PMC3215588

[B75] Iwamoto D. V. Calderwood D. A. (2015). Regulation of integrin-mediated adhesions. *Curr. Opin. Cell Biol.* 36 41–47. 10.1016/j.ceb.2015.06.009 26189062 PMC4639423

[B76] Johansson K. Törngren M. Wasselius J. Månsson L. Ehinger B. (2001). Developmental expression of DCC in the rat retina. *Dev. Brain Res.* 130 133–138. 10.1016/S0165-3806(01)00221-8 11557102

[B77] Kadamur G. Ross E. M. (2013). Mammalian phospholipase C. *Annu. Rev. Physiol.* 75 127–154. 10.1146/annurev-physiol-030212-183750 23140367

[B78] Kappler J. Franken S. Junghans U. Hoffmann R. Linke T. Müller H. W. (2000). Glycosaminoglycan-binding properties and secondary structure of the C-terminus of netrin-1. *Biochem. Biophys. Res. Commun.* 271 287–291. 10.1006/bbrc.2000.2583 10799289

[B79] Keino-Masu K. Masu M. Hinck L. Leonardo E. D. Chan S. S. Y. Culotti J. G. (1996). Deleted in colorectal cancer (DCC) encodes a netrin receptor. *Cell* 87 175–185. 10.1016/S0092-8674(00)81336-7 8861902

[B80] Keleman K. Dickson B. J. (2001). Short- and long-range repulsion by the Drosophila Unc5 Netrin receptor. *Neuron* 32 605–617. 10.1016/S0896-6273(01)00505-0 11719202

[B81] Kennedy T. E. Serafini T. de la Torre J. R. Tessier-Lavigne M. (1994). Netrins are diffusible chemotropic factors for commissural axons in the embryonic spinal cord. *Cell* 78 425–435. 10.1016/0092-8674(94)90421-9 8062385

[B82] Kennedy T. E. Wang H. Marshall W. Tessier-Lavigne M. (2006). Axon guidance by diffusible chemoattractants: a gradient of netrin protein in the developing spinal cord. *J. Neurosci.* 26 8866–8874. 10.1523/JNEUROSCI.5191-05.2006 16928876 PMC6674364

[B83] Kerstein P. C. Nichol R. H. Gomez T. M. (2015). Mechanochemical regulation of growth cone motility. *Front. Cell. Neurosci.* 9:244. 10.3389/fncel.2015.00244 26217175 PMC4493769

[B84] Killeen M. Tong J. Krizus A. Steven R. Scott I. Pawson T. (2002). UNC-5 function requires phosphorylation of cytoplasmic tyrosine 482, but its UNC-40-independent functions also require a region between the ZU-5 and death domains. *Dev. Biol.* 251 348–366. 10.1006/dbio.2002.0825 12435363

[B85] Kim T. H. Lee H. K. Seo I. A. Bae H. R. Suh D. J. Wu J. (2005). Netrin induces down-regulation of its receptor, deleted in colorectal cancer, through the ubiquitin-proteasome pathway in the embryonic cortical neuron. *J. Neurochem.* 95 1–8. 10.1111/j.1471-4159.2005.03314.x 16181408 PMC2683579

[B86] Kliesch T. T. Dietz J. Turco L. Halder P. Polo E. Tarantola M. (2017). Membrane tension increases fusion efficiency of model membranes in the presence of SNAREs. *Sci. Rep.* 7:12070. 10.1038/s41598-017-12348-w 28935937 PMC5608890

[B87] Kolodziej P. A. Timpe L. C. Mitchell K. J. Fried S. R. Goodman C. S. Jan L. Y. (1996). frazzled encodes a drosophila member of the DCC immunoglobulin subfamily and is required for CNS and motor axon guidance. *Cell* 87 197–204. 10.1016/S0092-8674(00)81338-0 8861904

[B88] Lanier L. M. Gates M. A. Witke W. Menzies A. S. Wehman A. M. Macklis J. D. (1999). Mena is required for neurulation and commissure formation. *Neuron* 22 313–325. 10.1016/S0896-6273(00)81092-2 10069337

[B89] Lebrand C. Dent E. W. Strasser G. A. Lanier L. M. Krause M. Svitkina T. M. (2004). Critical role of Ena/VASP proteins for filopodia formation in neurons and in function downstream of netrin-1. *Neuron* 42 37–49. 10.1016/S0896-6273(04)00108-4 15066263

[B90] Li W. Aurandt J. Jürgense C. Rao Y. Guan K.-L. (2006). FAK and Src kinases are required for netrin-induced tyrosine phosphorylation of UNC5. *J. Cell Sci.* 119 47–55. 10.1242/jcs.02697 16371650 PMC2248276

[B91] Li W. Lee J. Vikis H. G. Lee S.-H. Liu G. Aurandt J. (2004). Activation of FAK and Src are receptor-proximal events required for netrin signaling. *Nat. Neurosci.* 7 1213–1221. 10.1038/nn1329 15494734 PMC2373267

[B92] Li X. Gao X. Liu G. Xiong W. Wu J. Rao Y. (2008). Netrin signal transduction and the guanine nucleotide exchange factor DOCK180 in attractive signaling. *Nat. Neurosci.* 11 28–35. 10.1038/nn2022 18066058 PMC2262939

[B93] Li X. Meriane M. Triki I. Shekarabi M. Kennedy T. E. Larose L. (2002a). The adaptor protein Nck-1 couples the netrin-1 receptor DCC (deleted in colorectal cancer) to the activation of the small GTPase Racl through an atypical mechanism. *J. Biol. Chem.* 277 37788–37797. 10.1074/jbc.M205428200 12149262

[B94] Li X. Saint-Cyr-Proulx E. Aktories K. Lamarche-Vane N. (2002b). Rac1 and Cdc42 but not RhoA or Rho kinase activities are required for neurite outgrowth induced by the netrin-1 receptor DCC (deleted in colorectal cancer) in N1E-115 neuroblastoma cells. *J. Biol. Chem.* 277 15207–15214. 10.1074/jbc.M109913200 11844789

[B95] Liu G. Li W. Wang L. Kar A. Guan K.-L. Rao Y. (2009). DSCAM functions as a netrin receptor in commissural axon pathfinding. *Proc. Natl. Acad. Sci. U.S.A.* 106 2951–2956. 10.1073/pnas.0811083106 19196994 PMC2650370

[B96] Liu Y. Bhowmick T. Liu Y. Gao X. Mertens H. D. T. Svergun D. I. (2018). Structural basis for draxin-modulated axon guidance and fasciculation by netrin-1 through DCC. *Neuron* 97 1261.e–1267.e. 10.1016/j.neuron.2018.02.010 29503192 PMC5871715

[B97] Liu Y. Peng Y. Dai P.-G. Du Q.-S. Mei L. Xiong W.-C. (2012). Differential regulation of myosin X movements by its cargos, DCC and neogenin. *J. Cell Sci.* 125 751–762. 10.1242/jcs.094946 22349703 PMC3367835

[B98] Lucanic M. Kiley M. Ashcroft N. L’Etoile N. Cheng H.-J. (2006). The *Caenorhabditis elegans* P21-activated kinases are differentially required for UNC-6/netrin-mediated commissural motor axon guidance. *Development* 133 4549–4559. 10.1242/dev.02648 17050621

[B99] Ly A. Nikolaev A. Suresh G. Zheng Y. Tessier-Lavigne M. Stein E. (2008). DSCAM is a netrin receptor that collaborates with DCC in mediating turning responses to netrin-1. *Cell* 133 1241–1254. 10.1016/j.cell.2008.05.030 18585357 PMC2491333

[B100] Ma W. Shang Y. Wei Z. Wen W. Wang W. Zhang M. (2010). Phosphorylation of DCC by ERK2 is facilitated by direct docking of the receptor P1 domain to the kinase. *Structure* 18 1502–1511. 10.1016/j.str.2010.08.011 21070949

[B101] Mackay D. J. G. Esch F. Furthmayr H. Hall A. (1997). Rho- and Rac-dependent assembly of focal adhesion complexes and actin filaments in permeabilized fibroblasts: an essential role for ezrin/radixin/moesin proteins. *J. Cell Biol.* 138 927–938. 10.1083/jcb.138.4.927 9265657 PMC2138043

[B102] Maclennan A. J. Mclaurin D. L. Marks L. Vinson E. N. Pfeifer M. Szulc S. V. (1997). Immunohistochemical localization of netrin-1 in the embryonic chick nervous system. *J. Neurosci.* 17 5466–5479. 10.1523/JNEUROSCI.17-14-05466.1997 9204929 PMC6793803

[B103] Martín M. Simon-Assmann P. Kedinger M. Martin M. Mangeat P. Real F. X. (2006). DCC regulates cell adhesion in human colon cancer derived HT-29 cells and associates with ezrin. *Eur. J. Cell Biol.* 85 769–783. 10.1016/j.ejcb.2006.02.013 16762451

[B104] Masuda T. Sakuma C. Yaginuma H. (2009). Role for netrin-1 in sensory axonal guidance in higher vertebrates. *Fukushima J. Med. Sci.* 55 1–6. 10.5387/fms.55.1 19999164

[B105] Matsumoto H. Nagashima M. (2010). Netrin-1 elevates the level and induces cluster formation of its receptor DCC at the surface of cortical axon shafts in an exocytosis-dependent manner. *Neurosci. Res.* 67 99–107. 10.1016/j.neures.2010.02.004 20170691

[B106] Mehlen P. Furne C. (2005). Netrin-1: when a neuronal guidance cue turns out to be a regulator of tumorigenesis. *Cell. Mol. Life Sci.* 62 2599–2616. 10.1007/s00018-005-5191-3 16158190 PMC11139161

[B107] Mehlen P. Rama N. (2007). Nétrine-1 et guidage axonal: signalisation et traduction asymétrique. *Med. Sci.* 23 311–315. 10.1051/medsci/2007233311 17349294

[B108] Menon S. Boyer N. P. Winkle C. C. McClain L. M. Hanlin C. C. Pandey D. (2015). The E3 ubiquitin ligase TRIM9 is a filopodia off switch required for netrin dependent axon guidance. *Dev. Cell* 35 698–712. 10.1016/j.devcel.2015.11.022 26702829 PMC4707677

[B109] Meriane M. Tcherkezian J. Webber C. A. Danek E. I. Triki I. McFarlane S. (2004). Phosphorylation of DCC by Fyn mediates Netrin-1 signaling in growth cone guidance. *J. Cell Biol.* 167 687–698. 10.1083/jcb.200405053 15557120 PMC2172574

[B110] Millard S. S. Zipursky S. L. (2008). Dscam-mediated repulsion controls tiling and self-avoidance. *Curr. Opin. Neurobiol.* 18 84–89. 10.1016/j.conb.2008.05.005 18538559 PMC2707353

[B111] Mille F. Llambi F. Guix C. Delloye-Bourgeois C. Guenebeaud C. Castro-Obregon S. (2009). Interfering with multimerization of netrin-1 receptors triggers tumor cell death. *Cell Death Differ.* 16 1344–1351. 10.1038/cdd.2009.75 19543238 PMC2841642

[B112] Ming G. Song H. Berninger B. Inagaki N. Tessier-Lavigne M. Poo M. (1999). Phospholipase C-γ and phosphoinositide 3-kinase mediate cytoplasmic signaling in nerve growth cone guidance. *Neuron* 23 139–148. 10.1016/S0896-6273(00)80760-6 10402200

[B113] Ming G. L. Song H. J. Berninger B. Holt C. E. Tessier-Lavigne M. Poo M. (1997). cAMP-dependent growth cone guidance by netrin-1. *Neuron* 19 1225–1235. 10.1016/S0896-6273(00)80414-6 9427246

[B114] Montesinos M. L. (2014). “Roles for DSCAM and DSCAML1 in central nervous system development and disease,” in *Cell Adhesion Molecules: Implications in Neurological Diseases*, eds Berezin V. Walmod P. S. (New York, NY: Springer), 249–270. 10.1007/978-1-4614-8090-7_11 25300140

[B115] Moore S. W. Biais N. Sheetz M. P. (2009). Traction on immobilized netrin-1 is sufficient to reorient axons. *Science* 325:166. 10.1126/science.1173851 19589994 PMC2746731

[B116] Moore S. W. Correia J. P. Lai Wing Sun K. Pool M. Fournier A. E. Kennedy T. E. (2008). Rho inhibition recruits DCC to the neuronal plasma membrane and enhances axon chemoattraction to netrin 1. *Development* 135 2855–2864. 10.1242/dev.024133 18653556

[B117] Moore S. W. Kennedy T. E. (2006). Protein kinase A regulates the sensitivity of spinal commissural axon turning to netrin-1 but does not switch between chemoattraction and chemorepulsion. *J. Neurosci.* 26 2419–2423. 10.1523/JNEUROSCI.5419-05.2006 16510719 PMC6793650

[B118] Moore S. W. Tessier-Lavigne M. Kennedy T. E. (2007). “Netrins and their receptors,” in *Axon Growth and Guidance*, ed. Bagnard D. (New York, NY: Springer), 17–31. 10.1007/978-0-387-76715-4_2 18269208

[B119] Moore S. W. Zhang X. Lynch C. D. Sheetz M. P. (2012). Netrin-1 attracts axons through FAK-dependent mechanotransduction. *J. Neurosci.* 32 11574–11585. 10.1523/JNEUROSCI.0999-12.2012 22915102 PMC3461192

[B120] Morales D. (2018). A new model for netrin1 in commissural axon guidance. *J. Neurosci. Res.* 96 247–252. 10.1002/jnr.24117 28742927

[B121] Mori T. Kitano K. Terawaki S. I. Maesaki R. Fukami Y. Hakoshima T. (2008). Structural basis for CD44 recognition by ERM proteins. *J. Biol. Chem.* 283 29602–29612. 10.1074/jbc.M803606200 18753140 PMC2662033

[B122] Mullins R. D. (2000). How WASP-family proteins and the Arp2/3 complex convert intracellular signals into cytoskeletal structures. *Curr. Opin. Cell Biol.* 12 91–96. 10.1016/S0955-0674(99)00061-7 10679362

[B123] Murray A. Naeem A. Barnes S. H. Drescher U. Guthrie S. (2010). Slit and Netrin-1 guide cranial motor axon pathfinding via Rho-kinase, myosin light chain kinase and myosin II. *Neural Dev.* 5:16. 10.1186/1749-8104-5-16 20569485 PMC2907369

[B124] Nishiyama M. Hoshino A. Tsai L. Henley J. R. Goshima Y. Tessier-Lavigne M. (2003). Cyclic AMP/GMP-dependent modulation of Ca^2+^ channels sets the polarity of nerve growth-cone turning. *Nature* 423 990–995. 10.1038/nature01721.1 12827203

[B125] Norris A. D. Sundararajan L. Morgan D. E. Roberts Z. J. Lundquist E. A. (2014). The UNC-6/Netrin receptors UNC-40/DCC and UNC-5 inhibit growth cone filopodial protrusion via UNC-73/Trio, Rac-like GTPases and UNC-33/CRMP. *Development* 141 4395–4405. 10.1242/dev.110437 25371370 PMC4302909

[B126] Omotade O. F. Pollitt S. L. Zheng J. Q. (2017). Actin-based growth cone motility and guidance. *Mol. Cell. Neurosci.* 84 4–10. 10.1016/j.mcn.2017.03.001 28268126 PMC5587356

[B127] Palmesino E. Haddick P. C. G. Tessier-Lavigne M. Kania A. (2012). Genetic analysis of DSCAM’s role as a netrin-1 receptor in vertebrates. *J. Neurosci.* 32 411–416. 10.1523/JNEUROSCI.3563-11.2012 22238077 PMC6621089

[B128] Patterson R. L. Van Rossum D. B. Nikolaidis N. Gill D. L. Snyder S. H. (2005). Phospholipase C-γ: diverse roles in receptor-mediated calcium signaling. *Trends Biochem. Sci.* 30 688–697. 10.1016/j.tibs.2005.10.005 16260143

[B129] Phan K. D. Croteau L. P. Kam J. W. K. Kania A. Cloutier J. F. Butler S. J. (2011). Neogenin may functionally substitute for Dcc in chicken. *PLoS One* 6:e22072. 10.1371/journal.pone.0022072 21779375 PMC3133656

[B130] Picard M. Petrie R. J. Antoine-Bertrand J. Saint-Cyr-Proulx E. Villemure J. F. Lamarche-Vane N. (2009). Spatial and temporal activation of the small GTPases RhoA and Rac1 by the netrin-1 receptor UNC5a during neurite outgrowth. *Cell. Signal.* 21 1961–1973. 10.1016/j.cellsig.2009.09.004 19755150

[B131] Piperi C. Basdra E. K. (2015). Polycystins and mechanotransduction: from physiology to disease. *World J. Exp. Med.* 5 200–205. 10.5493/wjem.v5.i4.200 26618106 PMC4655249

[B132] Plooster M. Menon S. Winkle C. C. Urbina F. L. Monkiewicz C. Phend K. D. (2017). TRIM9-dependent ubiquitination of DCC constrains kinase signaling, exocytosis, and axon branching. *Mol. Biol. Cell* 28 2374–2385. 10.1091/mbc.E16-08-0594 28701345 PMC5576901

[B133] Pollard T. D. Blanchoin L. Mullins R. D. (2000). Molecular mechanisms controlling actin filament dynamics in nonmuscle cells. *Annu. Rev. Biophys. Biomol. Struct.* 29 545–576. 10940259 10.1146/annurev.biophys.29.1.545

[B134] Ponuwei G. A. (2016). A glimpse of the ERM proteins. *J. Biomed. Sci.* 23 4–9. 10.1186/s12929-016-0246-3 26983550 PMC4794931

[B135] Poullet P. Gautreau A. Kadaré G. Girault J. A. Louvard D. Arpin M. (2001). Ezrin interacts with focal adhesion kinase and induces its activation independently of cell-matrix adhesion. *J. Biol. Chem.* 276 37686–37691. 10.1074/jbc.M106175200 11468295

[B136] Purohit A. A. Li W. Qu C. Dwyer T. Shao Q. Guan K. L. (2012). Down syndrome cell adhesion molecule (DSCAM) associates with uncoordinated-5C (UNC5C) in netrin-1-mediated growth cone collapse. *J. Biol. Chem.* 287 27126–27138. 10.1074/jbc.M112.340174 22685302 PMC3411055

[B137] Qu C. Li W. Shao Q. Dwyer T. Huang H. Yang T. (2013). C-Jun N-terminal kinase 1 (JNK1) is required for coordination of netrin signaling in axon guidance. *J. Biol. Chem.* 288 1883–1895. 10.1074/jbc.M112.417881 23223444 PMC3548497

[B138] Quach T. T. Honnorat J. Kolattukudy P. E. Khanna R. Duchemin A. M. (2015). CRMPs: critical molecules for neurite morphogenesis and neuropsychiatric diseases. *Mol. Psychiatry* 20 1037–1045. 10.1038/mp.2015.77 26077693

[B139] Quiroga S. Bisbal M. Cáceres A. (2017). Regulation of plasma membrane expansion during axon formation. *Dev. Neurobiol.* 78 170–180. 10.1002/dneu.22553 29090510

[B140] Raber J. Olsen R. H. J. Su W. Foster S. Xing R. Acevedo S. F. (2014). CD44 is required for spatial memory retention and sensorimotor functions. *Behav. Brain Res.* 275 146–149. 10.1016/j.bbr.2014.09.010 25219362 PMC4253558

[B141] Ren X. R. Ming G. L. Xie Y. Hong Y. Sun D. M. Zhao Z. Q. (2004). Focal adhesion kinase in netrin-1 signaling. *Nat. Neurosci.* 7 1204–1212. 10.1038/nn1330 15494733

[B142] Ros O. Cotrufo T. Martínez-Mármol R. Soriano E. (2015). Regulation of patterned dynamics of local exocytosis in growth cones by netrin-1. *J. Neurosci.* 35 5156–5170. 10.1523/JNEUROSCI.0124-14.2015 25834042 PMC6705414

[B143] Rubtsov A. M. Lopina O. D. (2000). Ankyrins. *FEBS Lett.* 482 1–5. 11018513 10.1016/s0014-5793(00)01924-4

[B144] Russell S. A. Bashaw G. J. (2018). Axon guidance pathways and the control of gene expression. *Dev. Dyn.* 247 571–580. 10.1002/dvdy.24609 29226467 PMC6167058

[B145] Schmid R. S. Maness P. F. (2008). L1 and NCAM adhesion molecules as signaling coreceptors in neuronal migration and process outgrowth. *Curr. Opin. Neurobiol.* 18 245–250. 10.1016/j.conb.2008.07.015 18760361 PMC2633433

[B146] Sens P. Plastino J. (2015). Membrane tension and cytoskeleton organization in cell motility. *J. Phys. Condens. Matter* 27:273103. 10.1088/0953-8984/27/27/273103 26061624

[B147] Serafini T. Colamarino S. A. Leonardo E. D. Wang H. Beddington R. Skarnes W. C. (1996). Netrin-1 is required for commissural axon guidance in the developing vertebrate nervous system. *Cell* 87 1001–1014. 10.1016/S0092-8674(00)81795-X 8978605

[B148] Serafini T. Kennedy T. E. Gaiko M. J. Mirzayan C. Jessell T. M. Tessier-Lavigne M. (1994). The netrins define a family of axon outgrowth-promoting proteins homologous to *C. elegans* UNC-6. *Cell* 78 409–424. 10.1016/0092-8674(94)90420-0 8062384

[B149] Serrels B. Serrels A. Brunton V. G. Holt M. McLean G. W. Gray C. H. (2007). Focal adhesion kinase controls actin assembly via a FERM-mediated interaction with the Arp2/3 complex. *Nat. Cell Biol.* 9 1046–1056. 10.1038/ncb1626 17721515

[B150] Shao X. Q. Yang T. Huang H. Alarmanazi X. F. Liu X. G. (2017). Uncoupling of UNC5C with polymerized TUBB3 in microtubules mediates netrin-1 repulsion. *J. Neurosci.* 37 5620–5633. 10.1523/JNEUROSCI.2617-16.2017 28483977 PMC5469302

[B151] Shekarabi M. Kennedy T. E. (2002). The netrin-1 receptor DCC promotes filopodia formation and cell spreading by activating Cdc42 and Rac1. *Mol. Cell. Neurosci.* 19 1–17. 10.1006/mcne.2001.1075 11817894

[B152] Shekarabi M. Moore S. W. Tritsch N. X. Morris S. J. Bouchard J.-F. Kennedy T. E. (2005). Deleted in colorectal cancer binding netrin-1 mediates cell substrate adhesion and recruits Cdc42, Rac1, Pak1, and N-WASP into an intracellular signaling complex that promotes growth cone expansion. *J. Neurosci.* 25 3132–3141. 10.1523/JNEUROSCI.1920-04.2005 15788770 PMC6725078

[B153] Shu T. Valentino K. M. Seaman C. Cooper H. M. Richards L. J. (2000). Expression of the netrin-1 receptor, deleted in colorectal cancer (DCC), is largely confined to projecting neurons in the developing forebrain. *J. Comp. Neurol.* 416 201–212. 10581466 10.1002/(sici)1096-9861(20000110)416:2<201::aid-cne6>3.0.co;2-z

[B154] Solinet S. Mahmud K. Stewman S. F. Ben El Kadhi K. Decelle B. Talje L. (2013). The actin-binding ERM protein Moesin binds to and stabilizes microtubules at the cell cortex. *J. Cell Biol.* 202 251–260. 10.1083/jcb.201304052 23857773 PMC3718980

[B155] Stein E. Zou Y. Poo M. Tessier-Lavigne M. (2001). Binding of DCC by netrin-1 to mediate axon guidance independent of adenosine A2B receptor activation. *Science* 291 1976–1982. 10.1126/science.1059391 11239160

[B156] Suh Y. H. Chang K. Roche K. W. (2018). Metabotropic glutamate receptor trafficking. *Mol. Cell. Neurosci.* 10.1016/j.mcn.2018.03.014 [Epub ahead of print]. 29604330 PMC6128748

[B157] Sun K. L. W. Correia J. P. Kennedy T. E. (2011). Netrins: versatile extracellular cues with diverse functions. *Development* 138 2153–2169. 10.1242/dev.044529 21558366

[B158] Tadros W. Xu S. Akin O. Yi C. H. Shin G. J. Millard S. S. (2016). Dscam proteins direct dendritic targeting through adhesion. *Neuron* 89 480–493. 10.1016/j.neuron.2015.12.026 26844831 PMC4742784

[B159] Taylor A. M. Menon S. Gupton S. L. (2015). Passive microfluidic chamber for long-term imaging of axon guidance in response to soluble gradients. *Lab Chip* 15 2781–2789. 10.1039/C5LC00503E 26000554 PMC4485391

[B160] Terman J. R. Mao T. Pasterkamp R. J. Yu H. H. Kolodkin A. L. (2002). MICALs, a family of conserved flavoprotein oxidoreductases, function in plexin-mediated axonal repulsion. *Cell* 109 887–900. 10.1016/S0092-8674(02)00794-8 12110185

[B161] Tessier-Lavigne M. Placzek M. Lumsden A. G. S. Dodd J. Jessell T. M. (1988). Chemotropic guidance of developing axons in the mammalian central nervous system. *Nature* 336 775–778. 10.1038/336775a0 3205306

[B162] Tojima T. Akiyama H. Itofusa R. Li Y. Katayama H. Miyawaki A. (2007). Attractive axon guidance involves asymmetric membrane transport and exocytosis in the growth cone. *Nat. Neurosci.* 10 58–66. 10.1038/nn1814 17159991

[B163] Tojima T. Itofusa R. Kamiguchi H. (2014). Steering neuronal growth cones by shifting the imbalance between exocytosis and endocytosis. *J. Neurosci.* 34 7165–7178. 10.1523/JNEUROSCI.5261-13.2014 24849351 PMC6608195

[B164] Tong J. Killeen M. Steven R. Binns K. L. Culotti J. Pawson T. (2001). Netrin stimulates tyrosine phosphorylation of the UNC-5 family of netrin receptors and induces Shp2 binding to the RCM cytodomain. *J. Biol. Chem.* 276 40917–40925. 10.1074/jbc.M103872200 11533026

[B165] Urbina F. L. Gomez S. M. Gupton S. L. (2018). Spatiotemporal organization of exocytosis emerges during neuronal shape change. *J. Cell Biol.* 217 1113–1128. 10.1083/jcb.201709064 29351997 PMC5839795

[B166] Varadarajan S. G. Kong J. H. Phan K. D. Kao T. J. Panaitof S. C. Cardin J. (2017). Netrin1 produced by neural progenitors, not floor plate cells, is required for axon guidance in the spinal cord. *Neuron* 94 790.e–799.e. 10.1016/j.neuron.2017.03.007 28434801 PMC5576449

[B167] Wei Z. Yan J. Lu Q. Pan L. Zhang M. (2011). Cargo recognition mechanism of myosin X revealed by the structure of its tail MyTH4-FERM tandem in complex with the DCC P3 domain. *Proc. Natl. Acad. Sci. U.S.A.* 108 3572–3577. 10.1073/pnas.1016567108 21321230 PMC3048157

[B168] Wen Z. Guirland C. Ming G. Zheng J. Q. (2004). A CaMKII/calcineurin switch controls the direction of Ca^2+^ -dependent growth cone guidance. *Neuron* 43 835–846. 10.1016/j.neuron.2004.08.037 15363394

[B169] Williams M. E. Wu S. C.-Y. McKenna W. L. Hinck L. (2003). Surface expression of the netrin receptor UNC5H1 is regulated through a protein kinase C-interacting protein/protein kinase-dependent mechanism. *J. Neurosci.* 23 11279–11288. 10.1523/JNEUROSCI.23-36-11279.2003 14672991 PMC6740510

[B170] Wilson N. H. Key B. (2006). Neogenin interacts with RGMa and Netrin-1 to guide axons within the embryonic vertebrate forebrain. *Dev. Biol.* 296 485–498. 10.1016/j.ydbio.2006.06.018 16836993

[B171] Winckler B. Yap C. C. (2011). Endocytosis and endosomes at the crossroads of regulating trafficking of axon outgrowth-modifying receptors. *Traffic* 12 1099–1108. 10.1111/j.1600-0854.2011.01213.x 21535338 PMC3155643

[B172] Winkle C. C. McClain L. M. Valtschanoff J. G. Park C. S. Maglione C. Gupton S. L. (2014). A novel netrin-1-sensitive mechanism promotes local SNARE-mediated exocytosis during axon branching. *J. Cell Biol.* 205 217–232. 10.1083/jcb.201311003 24778312 PMC4003241

[B173] Xie Y. Hong Y. Ma X. Y. Ren X. R. Ackerman S. Mei L. (2006). DCC-dependent phospholipase C signaling in netrin-1-induced neurite elongation. *J. Biol. Chem.* 281 2605–2611. 10.1074/jbc.M512767200 16321979

[B174] Xu B. Goldman J. S. Rymar V. V. Forget C. Lo P. S. Bull S. J. (2010). Critical roles for the netrin receptor deleted in colorectal cancer in dopaminergic neuronal precursor migration, axon guidance, and axon arborization. *Neuroscience* 169 932–949. 10.1016/j.neuroscience.2010.05.025 20493932

[B175] Xu K. Wu Z. Renier N. Antipenko A. Tzvetkova-robev D. Xu Y. (2014). Structures of netrin-1 bound to two receptors provide insight into its axon guidance mechanism. *Science* 344 1275–1280. 10.1126/science.1255149 24876346 PMC4369087

[B176] Xu S. Liu Y. Li X. Liu Y. Meijers R. Zhang Y. (2018). The binding of DCC-P3 motif and FAK-FAT domain mediates the initial step of netrin-1/DCC signaling for axon attraction. *Cell Discov.* 4:8. 10.1038/s41421-017-0008-8 29479476 PMC5818605

[B177] Yamaguchi H. Shiraishi M. Fukami K. Tanabe A. Ikeda-Matsuo Y. Naito Y. (2009). MARCKS regulates lamellipodia formation induced by IGF-I via association with PIP2 and beta-actin at membrane microdomains. *J. Cell. Physiol.* 220 748–755. 10.1002/jcp.21822 19475567

[B178] Yamauchi K. Yamazaki M. Abe M. Sakimura K. Lickert H. Kawasaki T. (2017). Netrin-1 derived from the ventricular zone, but not the floor plate, directs hindbrain commissural axons to the ventral midline. *Sci. Rep.* 7:11992. 10.1038/s41598-017-12269-8 28931893 PMC5607380

[B179] Yang Z.-Z. Tschopp O. Baudry A. Dummler B. Hynx D. Hemmings B. A. (2004). Physiological functions of protein kinase B/Akt. *Biochem. Soc. Trans.* 32 393–396. 15046607 10.1042/bst0320350

[B180] Ylivinkka I. Keski-Oja J. Hyytiäinen M. (2016). Netrin-1: a regulator of cancer cell motility? *Eur. J. Cell Biol.* 95 513–520. 10.1016/j.ejcb.2016.10.002 27793362

[B181] Yung A. R. Nishitani A. M. Goodrich L. V. (2015). Phenotypic analysis of mice completely lacking netrin 1. *Development* 142 3686–3691. 10.1242/dev.128942 26395479 PMC4647218

[B182] Zhou J. Bronowska A. Le Coq J. Lietha D. Gräter F. (2015). Allosteric regulation of focal adhesion kinase by PIP2 and ATP. *Biophys. J.* 108 698–705. 10.1016/j.bpj.2014.11.3454 25650936 PMC4317530

[B183] Zhu K. Chen X. Liu J. Ye H. Zhu L. Wu J. Y. (2013). AMPK interacts with DSCAM and plays an important role in Netrin-1 induced neurite outgrowth. *Protein Cell* 4 155–161. 10.1007/s13238-012-2126-2 23479427 PMC3893083

[B184] Zhu X. J. Wang C. Z. Dai P. G. Xie Y. Song N. N. Liu Y. (2007). Myosin X regulates netrin receptors and functions in axonal path-finding. *Nat. Cell Biol.* 9 184–192. 10.1038/ncb1535 17237772

